# Diagnostic Accuracy of the HAS-BLED Bleeding Score in VKA- or DOAC-Treated Patients With Atrial Fibrillation: A Systematic Review and Meta-Analysis

**DOI:** 10.3389/fcvm.2021.757087

**Published:** 2021-11-22

**Authors:** Xinxing Gao, Xingming Cai, Yunyao Yang, Yue Zhou, Wengen Zhu

**Affiliations:** ^1^Division of Cardiology, Department of Internal Medicine, People's Hospital of Zhuzhou, Changsha Medical University, Zhuzhou, China; ^2^Department of Geriatric, The First Affiliated Hospital of Sun Yat-sen University, Guangzhou, China; ^3^Department of Cardiology, The First Affiliated Hospital of Sun Yat-sen University, Guangzhou, China; ^4^State Key Laboratory of Ophthalmology, Zhongshan Ophthalmic Center, Sun Yat-sen University, Guangzhou, China

**Keywords:** HAS-BLED, major bleeding, risk prediction, atrial fibrillation, meta-analysis

## Abstract

**Background:** Several bleeding risk assessment models have been developed in atrial fibrillation (AF) patients with oral anticoagulants, but the most appropriate tool for predicting bleeding remains uncertain. Therefore, we aimed to assess the diagnostic accuracy of the Hypertension, Abnormal liver/renal function, Stroke, Bleeding history or predisposition, Labile international normalized ratio, Elderly, Drugs/alcohol concomitantly (HAS-BLED) score compared with other risk scores in anticoagulated patients with AF.

**Methods:** We comprehensively searched the PubMed and Embase databases until July 2021 to identify relevant pieces of literature. The predictive abilities of risk scores were fully assessed by the C-statistic, net reclassification improvement (NRI) and integrated discrimination improvement (IDI) values, calibration data, and decision curve analyses.

**Results:** A total of 39 studies met the inclusion criteria. The C-statistic of the HAS-BLED score for predicting major bleeding was 0.63 (0.61–0.65) in anticoagulated patients regardless of vitamin k antagonists [0.63 (0.61–0.65)] and direct oral anticoagulants [0.63 (0.59–0.67)]. The HAS-BLED had the similar C-statistic to the Hepatic or renal disease, Ethanol abuse, Malignancy, Older, Reduced platelet count or function, Re-bleeding risk, Hypertension (uncontrolled), Anemia, Genetic factors, Excessive fall risk, Stroke (HEMORR_2_HAGES), the Anticoagulation and Risk Factors in Atrial Fibrillation (ATRIA), the Outcomes Registry for Better Informed Treatment of Atrial Fibrillation (ORBIT), the Global Anticoagulant Registry in the FIELD-Atrial Fibrillation (GARFIELD-AF), or the Age, Biomarkers, Clinical History (ABC) scores, but significantly higher C-statistic than the Congestive heart failure, Hypertension, Age ≥75 years, Diabetes mellitus, Stroke/transient ischemic attack history (CHADS_2_) or the Congestive heart failure/left ventricular ejection fraction ≤ 40%, Hypertension, Age ≥75 years, Diabetes mellitus, Stroke/transient ischemic attack/thromboembolism history, Vascular disease, Age 65–74 years, Sex (female) (CHA_2_DS_2_-VASc) scores. NRI and IDI values suggested that the HAS-BLED score performed better than the CHADS_2_ or the CHA_2_DS_2_-VASc scores and had similar or superior predictive ability compared with the HEMORR_2_HAGES, the ATRIA, the ORBIT, or the GARFIELD-AF scores. Calibration and decision curve analyses of the HAS-BLED score compared with other scores required further assessment due to the limited evidence.

**Conclusion:** The HAS-BLED score has moderate predictive abilities for bleeding risks in patients with AF regardless of type of oral anticoagulants. Current evidence support that the HAS-BLED score is at least non-inferior to the HEMORR_2_HAGES, the ATRIA, the ORBIT, the GARFIELD-AF, the CHADS_2_, the CHA_2_DS_2_-VASc, or the ABC scores.

## Introduction

Atrial fibrillation (AF), the most common cardiac arrhythmia in clinical practice, is associated with a 5-fold risk of ischemic stroke. Oral anticoagulation (OAC) is recommended to reduce the risk of thromboembolism among AF patients with the Congestive heart failure/left ventricular ejection fraction ≤ 40%, Hypertension, Age ≥75 years, Diabetes mellitus, Stroke/transient ischemic attack/thromboembolism history, Vascular disease, Age 65–74 years, Sex (female) (CHA_2_DS_2_-VASc) score of ≥2 in males or ≥3 in females (1). However, the use of OAC could increase the bleeding risks, especially major bleeding and intracranial bleeding, which are associated with increased rates of cardiovascular adverse events and death (2). Therefore, the risk assessment of bleeding after the initiation of OAC should be taken into consideration. The Hypertension, Abnormal liver/renal function, Stroke, Bleeding history or predisposition, Labile international normalized ratio, Elderly, Drugs/alcohol concomitantly (HAS-BLED) score is originated from the European Heart Survey database in 2010 (3), mainly focusing on the modifiable bleeding risk factors. The HAS-BLED score has been routinely recommended for predicting the bleeding risks in patients with AF who are taking anticoagulation (1).

In addition to the HAS-BLED score, several other bleeding risk assessment models have been developed in patients with AF (4–6). However, the differences in the predictive ability of the HAS-BLED score compared with other risk scores remain uncertain. Chang et al. (5) performed a network meta-analysis of 18 studies involving 321,888 patients and found that the HAS-BLED score was the most balanced bleeding risk prediction tool regarding sensitivity and specificity. Nevertheless, the sensitivity and specificity have limited guidance to clinicians when considering the probability of bleeding events in patients with AF (7). Zhu et al. (4) performed a meta-analysis including more critical measures (i.e., the C-statistic, reclassification, and calibration data), suggesting that the HAS-BLED score performed better for predicting major bleeding than other risk scores including the Hepatic or renal disease, Ethanol abuse, Malignancy, Older, Reduced platelet count or function, Re-bleeding risk, Hypertension (uncontrolled), Anemia, Genetic factors, Excessive fall risk, Stroke (HEMORR_2_HAGES), the Anticoagulation and Risk Factors in Atrial Fibrillation (ATRIA), the Congestive heart failure, Hypertension, Age ≥75 years, Diabetes mellitus, Stroke/transient ischemic attack history (CHADS_2_), and the CHA_2_DS_2_-VASc scores. However, in this study, they assessed the HAS-BLED score only in patients with AF with the use of vitamin k antagonists (VKAs). Since direct oral anticoagulants (DOACs) including dabigatran, rivaroxaban, apixaban, and edoxaban are recommended as the preferred drugs for stroke prevention among patients with non-valvular AF (1, 8), whether the application of the HAS-BLED score could be extended to DOAC-treated patients is still unclear. Therefore, this systematic review and meta-analysis aimed to: (1) assess the diagnostic accuracy of the HAS-BLED score in anticoagulated (VKAs or DOACs) patients with AF and (2) compared the performances of the HAS-BLED score with other risk scores to determine the most appropriate tool for predicting bleeding risks.

## Methods

This meta-analysis and systematic review were carried out based on the Cochrane Handbook for systemic reviews. The results were presented according to the Preferred Reporting Items for Systematic Reviews and Meta-Analyses (PRISMA) Statement. Ethical approval was not necessary because the published studies of electronic databases were included.

### Literature Search

We comprehensively conducted a search of the PubMed and Embase electronic databases until July 2021 to identify relevant pieces of literature reporting the HAS-BLED score in anticoagulated patients with AF. The following keywords in the search strategies were used: (1) AF, (2) VKAs or warfarin or coumadin or phenprocoumon or acenocoumarol or indandione or fluindione or phenindione or anisindione or non-VKAs or DOACs or dabigatran or rivaroxaban or apixaban or edoxaban, and (3) the HAS-BLED score. To obtain the qualified studies comprehensively, we also performed the cross-reference retrieval by screening the reference lists of included studies and prior meta-analyses. English language restrictions in the literature research were applied in this study.

### Eligibility Criteria

Studies were included if they met the following inclusion criteria: (a) patients with non-valvular AF (aged ≥ 18 years) with anticoagulants (VKAs or DOACs); (b) *post-hoc* analyses of randomized controlled trials (RCTs) or observational (prospective or retrospective cohorts) studies reported the diagnostic performance of the HAS-BLED score or focused on the predictive ability of the HAS-BLED score compared with any of other risk scores including the HEMORR_2_HAGES, the ATRIA, the ORBIT, the CHADS_2_, the CHA_2_DS_2_-VASc, the Global Anticoagulant Registry in the FIELD-Atrial Fibrillation (GARFIELD-AF), or Age, Biomarkers, Clinical History (ABC) (Supplementary Table 1); (c) the primary outcome was major bleeding and other bleeding events included any clinically relevant bleeding, any bleeding, intracranial bleeding, and gastrointestinal bleeding; and (d) at least one of the following data should be available: discrimination analysis (sensitivity/specificity or the C-statistic), net reclassification improvement (NRI) and integrated discrimination improvement (IDI) analyses, calibration data, and decision curve analyses. The sensitivity and specificity of the risk models have been analyzed by Chang et al. (5) in 2020 and, thus, were not included in this study.

We excluded studies that focused on the non-AF population or patients with AF with specific diseases (e.g., myocardial infarction, dialysis, ischemic stroke). In addition, we also excluded studies limiting to patients with AF with certain interventions (e.g., percutaneous coronary intervention, ablation). The bleeding risk prediction tools [e.g., the Rutherford score (9), mOBRI@@ (10), Adam score (11)] compared with the HAS-BLED score were not included, if they were analyzed for one bleeding endpoint in less than two independent studies. We also excluded studies reporting the modified HAS-BLED score version by adding additional factors (e.g., biomarkers, gene polymorphisms) into the original HAS-BLED score. Certain publication types with insufficient data such as reviews, case reports, editorials, or meeting abstracts were excluded.

### Study Selection and Data Extraction

Potentially relevant studies were selected by two reviewers independently based on the predetermined criteria. Qualified articles were included after the title/abstract screenings and the full-text screenings. At this step, if two or more studies had the same data source, we selected the study that was more designed to meet our inclusion criteria. If both the studies meet the inclusion criteria, we selected the newly published study or the study with the longest follow-up or highest sample size. Disagreements were resolved through discussion between the two researchers or consultation with a third reviewer.

Data from included studies were extracted by two researchers independently. We abstracted the following baseline information including the authors, year of publication, study type, data source, demographic data, baseline characteristics of the patient [age, sex ratio, sample size, type of anticoagulants, concomitant antiplatelet drugs, or non-steroidal anti-inflammatory drugs (NSAIDs)], study endpoints and their definitions, and the follow-up time.

### Quality Assessment

We applied the Prediction Model Risk of Bias Assessment Tool (PROBAST) (www.probast.org) to assess the risk prediction models (12). The PROBAST consists of four domains, namely participants, predictors, outcomes, and analysis. Risk assessment was rated as low risk, high risk, or unclear.

### Consistency Test and Publication Bias

The consistency of the included studies was assessed through the Cochrane *Q* test and *I*^2^ index. Significant heterogeneity was considered if the *p*-value of the Cochrane *Q* test < 0.1 or if the *I*^2^ value of >50%. We used the funnel plots to examine the publication bias and a visual inspection of asymmetry indicated a bias.

### Statistical Analysis

All the statistical analyses were carried out by using the Review Manager 5.3 software (The Nordic Cochrane Center, The Cochrane Collaboration, 2014, Copenhagen, Denmark, UK) (https://community.cochrane.org/). *p* < 0.05 was considered as statistically significant.

The C-statistic and their 95% CIs were abstracted from the included studies for the discrimination analysis. The C-statistic of ≤ 0.5 indicated that discrimination was no better than chance. The pooled analyses were performed if at least two studies reported the C-statistic for the HAS-BLED score. A random-effects model with an inverse variance method was chosen in the pooled analysis due to the observed significant heterogeneity. For the primary major bleeding events, the subgroup analyses were conducted on the basis of study design, the OAC type, and the follow-up time. We also assessed the predictive ability of the HAS-BLED based on available vs. unavailable labile international normalized ratio (INR) in the score.

The Z-statistic was calculated to compare the two C-statistic of the HAS-BLED score vs. other risk prediction models (the HEMORR_2_HAGES, the ATRIA, the ORBIT, the CHADS_2_, the CHA_2_DS_2_-VASc, the GARFIELD-AF, or the ABC scores) (4). In addition, we assessed the improvement in predictive accuracy by the reclassification analyses including the NRI and IDI parameters. The probability of correctly predicting bleeding events by using the HAS-BLED score was reflected in the percentage of events correctly reclassified. Calibration data represented the extent to which predicted risks correspond to observed risks. The net benefits of the HAS-BLED vs. other risk scores were assessed by using the decision curve analyses. Narrative analyses were performed with respect to reclassification, calibration, and decision curve analyses due to the lack of numerical data.

## Results

### Study Selection

The flowchart of document retrieval and screening process in this meta-analysis is shown in Supplementary Figure 1. We initially retrieved 1,601 studies through the electronic search of the PubMed and the Embase databases. After the screenings of the titles and abstracts, 97 studies were assessed for more detail. Furthermore, 58 of these studies were excluded because: (1) patients with OACs were not analyzed separately (*n* = 5); (2) duplicate data (*n* = 9); (3) the anticoagulated drugs were not VKAs or DOACs or unknown OAC data (*n* = 8); (4) studies did not report the C-statistic and/or their CIs (*n* = 8); (5) non-AF population or AF patients with coexisting specific diseases (*n* = 25); and (6) the outcome of bleeding was not analyzed separately (*n* = 3). Finally, a total of 39 studies published from 2010 to 2021 met the inclusion criteria and were included in this study (Supplementary Table 2).

The baseline characteristics of patient of the included studies are summarized in Table 1. The variables of the HAS-BLED score in the included studies are presented in Supplementary Table 3. The component of “labile INR” in the HAS-BLED score was available in 10 included studies. “Labile INR” was not applicable in three studies (13–15) because they only included DOAC-treated patients for analysis. As shown in Supplementary Table 4, all the included studies had high (*n* = 20, 51%) or unclear (*n* = 19, 49%) risk of bias according to the PROBAST tool.

**Table 1:** Baseline characteristics of patient of the included studies.

**Study (author-year)**	**Data source**	**Study design**	Sample size^*^	**Type of anticoagulants analyzed**	**Concomitant antiplatelet or NSAIDS**	**Study endpoints**	**Bleeding scales**	**Comparisons of HAS-BLED vs. others**	**Follow-up time (y)**
Pisters-2010	Euro Heart Survey on AF; 2003–2004	Retrospective cohort	1,722	VKAs	NA	Major bleeding	ISTH	HEMORR2HAGES	1.0
Olesen-2011	Danish National Patient Registry; 1997–2006	Retrospective cohort	44,771	VKAs (99.8%), heparins	33%	Major bleeding	ICD codes	HEMORR2HAGES	1.0
Friberg-2012	Swedish National Hospital Discharge Registry; 2005–2008	Retrospective cohort	48,599	Warfarin	NA	Major bleeding; Intracranial bleeding	ICD codes	HEMORR2HAGES	1.50
Apostolakis-2012	The AMADEUS trial	*Post-hoc* analysis of RCT	2,293	Warfarin	18%	Major bleeding; Any clinically relevant bleeding	ISTH	HEMORR2HAGES; ATRIA	1.18
Apostolakis-2013	The AMADEUS trial	*Post-hoc* analysis of RCT	2,293	Warfarin	18%	Any clinically relevant bleeding	ISTH	CHADS2; CHA2DS2-VASc	1.18
Senoo-2016	The AMADEUS trial	*Post-hoc* analysis of RCT	2,293	Warfarin	16.5%	Major bleeding; Any clinically relevant bleeding	ISTH	ORBIT	1.18
Proietti-2016	The SPORTIF III and V clinical trials	*Post-hoc* analysis of RCT	3,551	Warfarin	19.9%	Major bleeding	ISTH	ORBIT; ATRIA; HEMORRAGES	1.6
Proietti-2018a	The SPORTIF III clinical trial	*Post-hoc* analysis of RCT	3,550	Warfarin	19.9%	Major bleeding; Any clinically relevant bleeding; Any bleeding	ISTH	GARFIELD-AF	1.56
Roldan-2013a	Outpatient anticoagulation clinic; City Hospital, Birmingham, UK; 2007.03–2008.11	Prospective cohort	937	Acenocoumarol	17%	Major bleeding	ISTH	ATRIA	2.61
Roldan-2013b	Outpatient anticoagulation clinic; City Hospital, Birmingham, UK; 2007–2008	Prospective cohort	1,370	Acenocoumarol	18%	Major bleeding	ISTH	CHADS2; CHA2DS2-VASc	2.73
Barnes-2014	Michigan Anticoagulation Quality Improvement Initiative (MAQI2); 2009–2012	Prospective cohort	2,600	Warfarin	NA	Major bleeding	ISTH	HEMORR2HAGES; ATRIA; CHADS2; CHA2DS2-VASc	1.0
Esteve-Pastor-2016	The FANTASIIA registry; Spanish; 2013–2014	Prospective cohort	1,276	DOACs; VKAs	10.9%	Major bleeding	ISTH	ORBIT	1.0
Hijazi-2016	The ARISTOTLE derivation cohort	*Post-hoc* analysis of RCT	14,537	Apixaban; warfarin	39%	Major bleeding	ISTH	ABC; ORBIT	1.9
	The RE-LY validation cohort	*Post-hoc* analysis of RCT	8,468	Dabigatran; warfarin	44%	Major bleeding	ISTH	ABC; ORBIT	1.9
Proietti-2018b	The RE-LY trial, whole cohort	*Post-hoc* analysis of RCT	18,113	Dabigatran; warfarin	40%	Major bleeding; Intracranial bleeding	ISTH	HEMORR2HAGES; ATRIA; ORBIT	2.0
Berg-2019	The ENGAGE AF-TIMI 48 trial	*Post-hoc* analysis of RCT	8,705	Edoxaban; Warfarin	NA	Major bleeding	ISTH	ABC	2.8
Jaspers Focks-2016	The anticoagulation clinic in the region Arnhem/Nijmegen, the Netherlands	Prospective cohort	1,157	VKAs (acenocoumarol 90%)	4.1%	Major bleeding; Any clinically relevant bleeding; Any bleeding	ISTH	HEMORR2HAGES; ATRIA	2.5
Steinberg-2016	The ORBIT-AF registry; US outpatients	Prospective cohort	7,420	Dabigatran; Warfarin	NA	Major bleeding	ISTH	ATRIA	NA
Poli-2017	The START register, multicenter in Italy	Prospective cohort	4,579	DOACs; VKAs	16.5%	Major bleeding	ISTH	CHADS2; CHA2DS2-VASc	1.4
Caro Martínez-2017	Three Spanish hospitals; 2013–2014	Retrospective cohort	973	DOACs	NA	Major bleeding; Gastrointestinal bleeding	ISTH	ATRIA; ORBIT	1.77
Elvira-Ruiz-2020	Two hospitals in Spain; 2013–2016	Retrospective cohort	2,880	DOACs; VKAs	17.7%	Major bleeding	ISTH	ATRIA; ORBIT	1.5
Esteve-Pastor-2017	Single anticoagulation centre in a tertiary hospital in Murcia, Spain; 2007	Prospective cohort	1,120	Acenocoumarol	NA	Major bleeding; Intracranial bleeding; Gastrointestinal bleeding	ISTH	ABC	6.5
Rivera-Caravaca-2017	Single anticoagulation centre in a tertiary hospital in Murcia, Spain; 2007	Retrospective cohort	1,361	Acenocoumarol	18%	Major bleeding	ISTH	HEMORR2HAGES; ATRIA; ORBIT	6.5
Fox-2021	The GARFIELD-AF registry from 35 countries; 2010–2016	Retrospective cohort	52,032	DOACs; VKAs	>12%	Major bleeding	ISTH	GARFIELD-AF	2.0
Beshir-2018	University of Malaya Medical Centre and Institut Jantung Negara or the National Heart Institute of Malaysia	Retrospective cohort	1,017	Warfarin, rivaroxaban, dabigatran	35%	Major bleeding; Clinically relevant non-major bleeding	ISTH	HEMORR 2HAGES, ATRIA; ORBIT	1.0
Chao-2018	National Health Insurance Research Database, Taiwan; 1998–2011	Retrospective cohort	40,450	Warfarin	22.7%	Major bleeding; Intracranial bleeding	NA	HEMORR2HAGES; ATRIA; ORBIT	4.6
Dalgaard-2019	Danish nationwide databases	Retrospective cohort	51,180	DOACs; VKAs	NA	Major bleeding	ICD codes	GARFIELD-AF	1.0
Lip-2018	Danish nationwide databases	Retrospective cohort	57,930	DOACs	39.1%	Any bleeding	ICD codes	ATRIA; ORBIT	2.5
Mori-2019	The DIRECT registry; single-center in Japan	Prospective cohort	2,216	DOACs	21.5%	Major bleeding	ISTH	ORBIT	0.86
O'Brien-2015	The ORBIT-AF registry; 176 sites in the USA	Prospective cohort	7,411	Dabigatran; Warfarin	37.9%	Major bleeding	ISTH	ATRIA; ORBIT	2.0
	The ROCKET-AF validation cohort	*Post-hoc* analysis of RCT	14,264	Warfarin, rivaroxaban	NA	Major bleeding	ISTH	ATRIA; ORBIT	1.9
Quinn-2016	The ATRIA Study; California; 1996–1997	Retrospective cohort	13,559	Warfarin	NA	Major bleeding	ISTH	CHADS2; CHA2DS2-VASc; ATRIA	NA
Yao-2017	OptumLabs Data Warehouse; US; 2010–2015	Retrospective cohort	39,539	DOACs	7%	Major bleeding; Intracranial bleeding	NA	CHADS2; CHA2DS2-VASc	0.6
Claxton-2018	The derivation (MarketScan, 2007–2014) and validation (Optum Clinformatics, 2009–2015) cohorts	Prospective cohort	81,285	DOACs; Warfarin	NA	Major bleeding	ISTH	HEMORR2HAGES; ATRIA; ORBIT	1.0
Rutherford-2018	Norwegian Patient Registry and Norwegian Prescription Database; 2013–2015	Retrospective cohort	21,248	DOACs	52.8%	Any clinically relevant bleeding	ICD codes	ATRIA; ORBIT	0.5
Adam-2021	Multicenter cohort study in Switzerland (SWISS-AF)	Prospective cohort	2,147	DOACs; VKAs	18%	Any clinically relevant bleeding	ISTH	ATRIA; ORBIT	4.4
Siu-2014	Queen Mary Hospital, Hong Kong; 1997–2011	Retrospective cohort	1,912	Warfarin	NA	Intracranial bleeding	NA	NA	3.19
Suzuki-2014	Kameda Medical Center; Japan; 2005	Prospective cohort	231	Warfarin	36.9–50%	Major bleeding	ISTH	NA	7.1
Prochaska-2018	The thrombEVAL cohort. Denmark	Prospective cohort	1,089	Phenprocoumon	37.9%	Any clinically relevant bleeding	NA	NA	3.0
Schwartz-2019	Northwestern Healthcare system's Enterprise Database Warehouse; US; 2011–2017	Retrospective cohort	9,819	DOACs; VKAs	NA	Major bleeding	ISTH	NA	1.84
Ravvaz-2021	Longitudinal electronic health records in eastern Wisconsin and northern Illinois	Retrospective cohort	7,274	Warfarin	NA	Any bleeding	ICD codes	NA	0.93

* *Number of anticoagulated patients*.

*HAS-BLED, Hypertension, Abnormal liver/renal function, Stroke, Bleeding history or predisposition, Labile international normalized ratio, Elderly, Drugs/alcohol concomitantly; HEMORR_2_HAGES, Hepatic or renal disease, Ethanol abuse, Malignancy, Older, Reduced platelet count or function, Re-bleeding risk, Hypertension (uncontrolled), Anemia, Genetic factors, Excessive fall risk, Stroke; ATRIA, Anticoagulation and Risk Factors in Atrial Fibrillation; ORBIT, Outcomes Registry for Better Informed Treatment of Atrial Fibrillation; GARFIELD-AF, Global Anticoagulant Registry in the FIELD-Atrial Fibrillation; ABC, Age, Biomarkers, Clinical History; CHADS_2_, Congestive heart failure, Hypertension, Age ≥ 75 years, Diabetes mellitus, Stroke/transient ischemic attack history; CHA_2_DS_2_-VASc, Congestive heart failure/left ventricular ejection fraction ≤ 40%, Hypertension, Age ≥ 75 years, Diabetes mellitus, Stroke/transient ischemic attack/thromboembolism history, Vascular disease, Age 65–74 years, Sex (female); ICD, International Classification of Diseases; ISTH, International Society of Thrombosis and Haemostasis; NSAIDS, non-steroidal anti-inflammatory drugs; NA, not available*.

### Diagnostic Accuracy of the HAS-BLED Score

**Figure 1:**
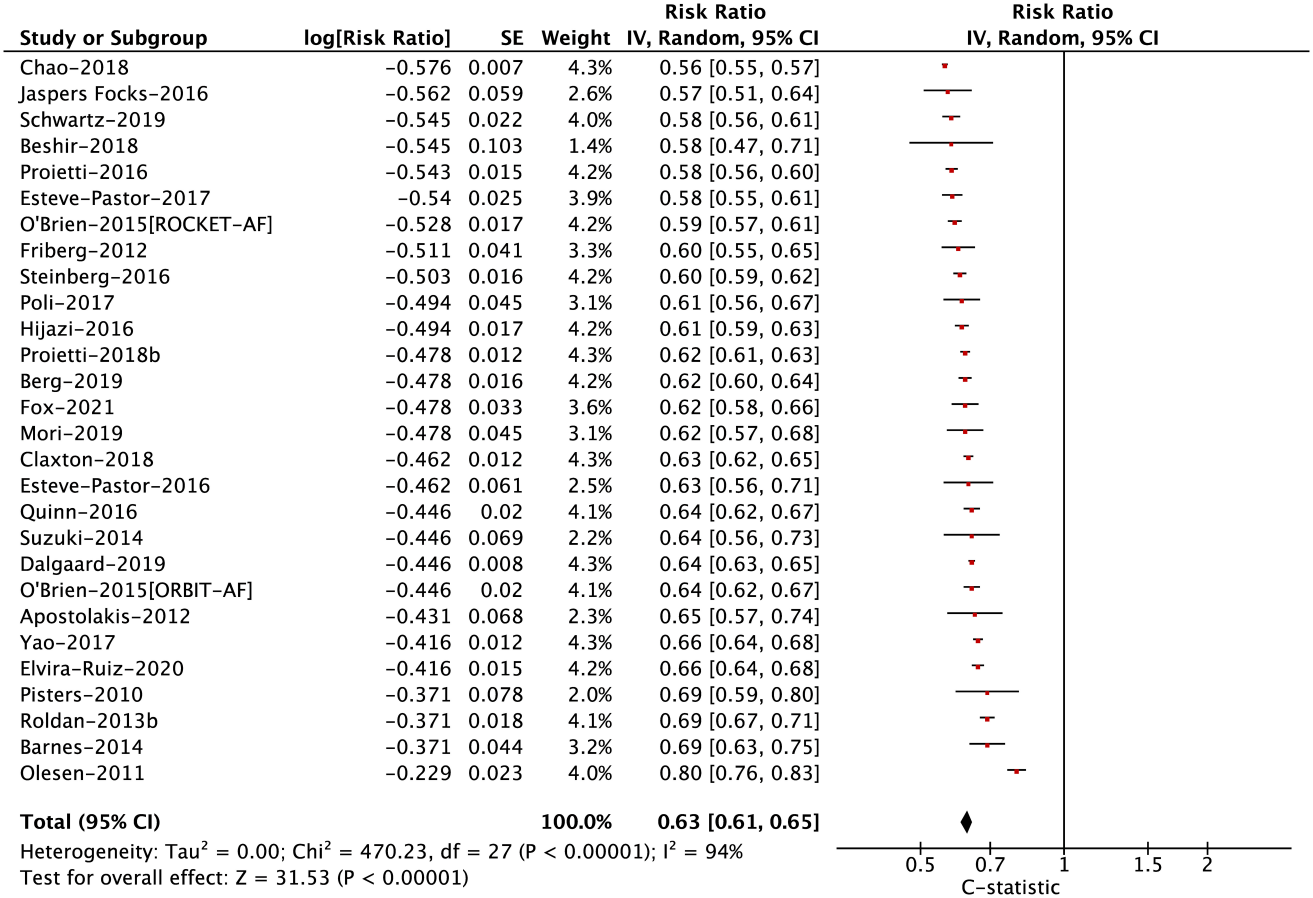
The pooled analysis of the C-statistic for major bleeding in anticoagulated patients with atrial fibrillation.

In anticoagulated patients with AF, the C-statistic for the HAS-BLED score ranged from 0.56 to 0.80 for major bleeding (median 0.62), 0.53 to 0.62 for any clinically relevant bleeding (median 0.58), 0.51 to 0.64 for any bleeding (median 0.57), 0.53 to 0.64 for intracranial bleeding (median 0.57), and 0.61 to 0.74 for gastrointestinal bleeding (median 0.68). In the pooled analysis, the C-statistic for major bleeding, any clinically relevant bleeding, any bleeding, intracranial bleeding, and gastrointestinal bleeding were 0.63 (0.61–0.65), 0.58 (0.56–0.61), 0.57 (0.53–0.61), 0.58 (0.53–0.62), and 0.67 (0.55–0.82), respectively (Figure 1; Supplementary Figure 2). For this part, there were no potential publication biases when inspecting the funnel plots (Supplementary Figure 3).

**Figure 2:**
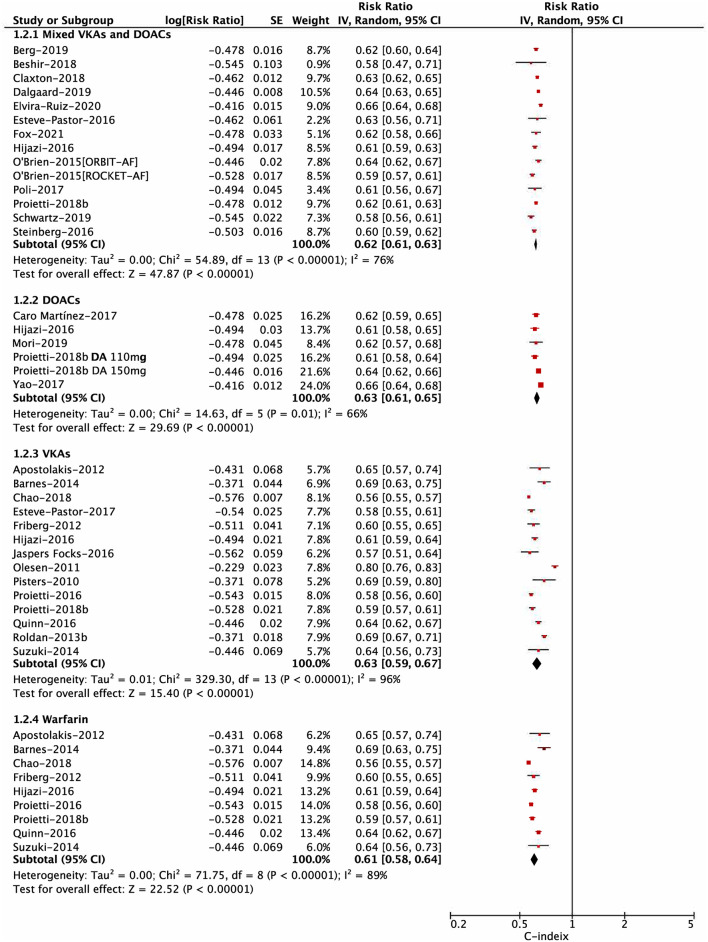
Subgroup analysis for pooling the C-statistic of the Hypertension, Abnormal liver/renal function, Stroke, Bleeding history or predisposition, Labile international normalized ratio, Elderly, Drugs/alcohol concomitantly (HAS-BLED) score based on the types of oral anticoagulation.

For the risk of major bleeding, in the subgroup analysis based on the OAC type shown in Figure 2, the C-statistic for major bleeding in four subgroups of mixed DOACs or VKAs, DOACs, VKAs, and warfarin were 0.62 (0.61–0.63), 0.63 (0.61–0.65), 0.63 (0.59–0.67), and 0.61 (0.58–0.64), respectively (*P*_interaction_ = 0.58). The subgroup analysis based on study design (*post-hoc* analysis of RCT, prospective cohort, and retrospective cohort) indicated no interaction between them (*P*_interaction_ = 0.14; Supplementary Figure 4). In addition, the predictive ability for major bleeding was also similar between available vs. unavailable labile INR in the HAS-BLED score (*P*_interaction_ = 0.19; Supplementary Figure 5). However, there was a difference in the subgroup analysis based on the follow-up time, suggesting that the HAS-BLED score performed better in the group of ≤ 12 months compared with that of >12 months (*P*_interaction_ = 0.01; Supplementary Figure 6).

### Performances of the HAS-BLED Score With Other Risk Scores

#### Discrimination Analysis

As shown in Table 2, for comparisons of the C-statistic between two different risk scores, there were no statistically significant differences between the HAS-BLED *vs*. the HEMORR_2_HAGES scores (major bleeding: Z-statistic = 0.396; any clinically relevant bleeding: 0.321; intracranial bleeding: −0.408); *vs*. the ORBIT (major bleeding: −0.911; any clinically relevant bleeding: 0; intracranial bleeding: −0.158); *vs*. the ATRIA (major bleeding −0.502; any clinically relevant bleeding: 0.257; intracranial bleeding: 0); *vs*. the GARFIELD-AF (major bleeding: −0.448); or *vs*. the ABC scores (major bleeding: −1.09) (*p* > 0.05), suggesting similar discrimination performances. However, the HAS-BLED score had significantly higher C-statistic for predicting major bleeding than the CHADS_2_ (Z-statistic = 2.19, *p* < 0.05) or the CHA_2_DS_2_-VASc scores (Z-statistic = 1.99, *p* < 0.05), suggesting that the HAS-BLED score performed better than the CHADS_2_ or CHA_2_DS_2_-VASc scores.

**Table 2:** Summary of the C-statistic and 95% CIs of the included studies.

	**Major bleeding**	**Any clinically relevant bleeding**	**Intracranial bleeding**
**Overall**			
No. of studies	28	7	6
C-statistic: HAS-BLED	0.63 [0.61, 0.65]	0.58 [0.56, 0.61]	0.58 [0.53, 0.62]
**HAS-BLED vs. HEMORR**_**2**_ **HAGES**			
No. of studies	12	3	3
C-statistic: HAS-BLED	0.63 [0.60, 0.67]	0.57 [0.54, 0.61]	0.56 [0.51, 0.63]
C-statistic: HEMORR2HAGES	0.62 [0.58, 0.65]	0.56 [0.51, 0.61]	0.58 [0.51, 0.66]
Z-statistic	0.396^#^	0.321^#^	−0.408^#^
**HAS-BLED vs. ORBIT**			
No. of studies	12	4	2
C-statistic: HAS-BLED	0.61 [0.59, 0.64]	0.59 [0.56, 0.63]	0.54 [0.51, 0.57]
C-statistic: ORBIT	0.63 [0.60, 0.67]	0.59 [0.53, 0.66]	0.55 [0.45, 0.69]
Z-statistic	−0.911^#^	0^#^	−0.158^#^
**HAS-BLED vs. ATRIA**			
No. of studies	15	5	2
C-statistic: HAS-BLED	0.62 [0.60, 0.65]	0.59 [0.56, 0.62]	0.54 [0.51, 0.57]
C-statistic: ATRIA	0.63 [0.60, 0.66]	0.58 [0.51, 0.65]	0.54 [0.47, 0.62]
Z-statistic	−0.502^#^	0.257^#^	0^#^
**HAS-BLED vs. CHADS2**			
No. of studies	5	-	-
C-statistic: HAS-BLED	0.66 [0.64, 0.68]	-	-
C-statistic: CHADS2	0.61 [0.57, 0.65]	-	-
Z-statistic	2.19^*^	-	-
**HAS-BLED vs. CHA2DS2-VASc**			
No. of studies	5	-	-
C-statistic: HAS-BLED	0.66 [0.64, 0.68]	-	-
C-statistic: CHA2DS2-VASc	0.61 [0.57, 0.66]	-	-
Z-statistic	1.99^*^	-	-
**HAS-BLED vs. GARFIELD-AF**			
No. of studies	3	-	-
C-statistic: HAS-BLED	0.61 [0.57, 0.66]	-	-
C-statistic: GARFIELD-AF	0.63 [0.56, 0.71]	-	-
Z-statistic	−0.448^#^	-	-
**HAS-BLED vs. ABC**			
No. of studies	4	-	-
C-statistic: HAS-BLED	0.61 [0.60, 0.63]	-	-
C-statistic: ABC	0.65 [0.58, 0.72]	-	-
Z-statistic	−1.09^#^	-	-

*The absolute value of Z-statistic more than 1.96 indicated a p-value of < 0.05, suggesting a significant difference in the discrimination between the two risk scores*.

* *p < 0.05*;

# *p > 0.05*.

*HAS-BLED, Hypertension, Abnormal liver/renal function, Stroke, Bleeding history or predisposition, Labile international normalized ratio, Elderly, Drugs/alcohol concomitantly; HEMORR_2_HAGES, Hepatic or renal disease, Ethanol abuse, Malignancy, Older, Reduced platelet count or function, Re-bleeding risk, Hypertension (uncontrolled), Anemia, Genetic factors, Excessive fall risk, Stroke; ATRIA, Anticoagulation and Risk Factors in Atrial Fibrillation; ORBIT, Outcomes Registry for Better Informed Treatment of Atrial Fibrillation; GARFIELD-AF, Global Anticoagulant Registry in the FIELD-Atrial Fibrillation; ABC, Age, Biomarkers, Clinical History; CHADS_2_, Congestive heart failure, Hypertension, Age ≥ 75 years, Diabetes mellitus, Stroke/transient ischemic attack history; CHA_2_DS_2_-VASc, Congestive heart failure/left ventricular ejection fraction ≤ 40%, Hypertension, Age ≥ 75 years, Diabetes mellitus, Stroke/transient ischemic attack/thromboembolism history, Vascular disease, Age 65–74 years, Sex (female)*.

#### Reclassification Analysis

As presented in Table 3, the HAS-BLED score for predicting major bleeding had both the significantly positive NRI and IDI values compared with the CHADS_2_ (16–18) or the CHA_2_DS_2_-VASc scores (13, 16, 17), suggesting that the predictive ability of the HAS-BLED score was more dominant than the CHADS_2_ or the CHA_2_DS_2_-VASc scores. The HAS-BLED score compared with the HEMORR_2_HAGES (19–22), the ATRIA (16, 19–23), or the ORBIT score (20–22) had positive NRI and IDI values, although non-significant in some studies (19, 21). Only one study of Jaspers Focks et al. (24) reported non-significant negative NRI values between the HAS-BLED vs. the HEMORR_2_HAGES or the ATRIA scores. In the study of Proietti et al. (25), the GARFIELD-AF compared with the HAS-BLED scores had both non-significant negative NRI and IDI values. Overall, the HAS-BLED score had at least non-inferior predictive ability for predicting major bleeding compared with the HEMORR_2_HAGES, the ATRIA, the ORBIT, or the GARFIELD-AF scores.

**Table 3:** NRI and IDI analysis for predicting the bleeding risks in anticoagulated patients with AF.

**Study (author-year)**	**NRI analysis**	**IDI analysis**
**Major bleeding**		
Apostolakis-2012	HAS-BLED vs. HEMORR_2_HAGES (+6.8%, *P* = 0.42); HAS-BLED vs. ATRIA(+9.0%, *P* = 0.33)	Not available
Roldan-2013a	Continuous: HAS-BLED vs. ATRIA(+13.6%, *P* = 0.43)	Continuous: HAS-BLED vs. ATRIA(+6.9%, *P* = 0.033)
	Categorical: HAS-BLED vs. ATRIA(+19.6%, *P* = 0.019)	Categorical: HAS-BLED vs. ATRIA(+7.0%, *P* = 0.001)
Roldan-2013b	HAS-BLED vs. CHADS_2_ (+38.62%, *P* < 0.001); HAS-BLED vs. CHA_2_DS_2_-VASc (+37.6%, *P* <0.001)	HAS-BLED vs. CHADS_2_ (+10.0%, *P* < 0.001);HAS-BLED vs. CHA_2_DS_2_-VASc (+12.0%, *P* < 0.001)
Barnes-2014	HAS-BLED vs. HEMORR_2_HAGES (+26.0%, *P* = 0.006); HAS-BLED vs. ATRIA (+31.0%, *P* = 0.001); HAS-BLED vs. CHADS_2_ (+58.0%, *P* < 0.001); HAS-BLED vs. CHA_2_DS_2_-VASc (+36.0%, *P* < 0.001)	Not available
Berg-2019	ABC vs. HAS-BLED +13.8% (8.0–22.8%)	Not available
Chao-2018	HAS-BLED vs. HEMORR_2_HAGES (+4.3%, *P* < 0.001); HAS-BLED vs. ATRIA (+4.9%, *P* < 0.001); HAS-BLED vs. ORBIT (+5.5%, *P* < 0.001)	Not available
Esteve-Pastor-2017	ABC vs. HAS-BLED (−13.74%, *P* = 0.005)	ABC vs. HAS-BLED (−13.14%, *P* = 0.002)
Jaspers Focks-2016	HAS-BLED vs. HEMORR_2_HAGES (−3.60%, *P* = 0.460); HAS-BLED vs. ATRIA (−6.32%, *P* = 0.894)	Not available
Proietti-2016	ORBIT vs. HAS-BLED (-0.77%, *P* = 0.392); ATRIA vs. HAS-BLED (-8.83%, *P* = 0.323); HEMORR2HAGES vs. HAS-BLED (−13.66%, *P* = 0.119)	ORBIT vs. HAS-BLED (0%, *P* = 0.646);ATRIA vs. HAS-BLED (0%, *P* = 0.611);HEMORR2HAGES vs. HAS-BLED (−0.18%, *P* = 0.039)
Proietti-2018a	GARFIELD vs. HAS-BLED (−4.2%, *P* = 0.448)	GARFIELD vs. HAS-BLED (−0.2%, *P* = 0.318)
Rivera-Caravaca-2017	HAS-BLED vs. HEMORR_2_HAGES (+15.74%, *P* < 0.001); HAS-BLED vs. ATRIA (+15.98%, *P* < 0.001); HAS-BLED vs. ORBIT (+12.12%, *P* = 0.007)	HAS-BLED vs. HEMORR_2_HAGES (+3.11%, *P* = 0.347);HAS-BLED vs. ATRIA (+3.09%, *P* = 0.142);HAS-BLED vs. ORBIT (+2.4%, *P* = 0.067)
Quinn-2016	HAS-BLED vs. CHADS_2_ (+0.4%)	Not available
Yao-2017	HAS-BLED vs. CHA_2_DS_2_-VASc (+2.0%, *P* < 0.001)	Not available
**Any clinically relevant bleeding**		
Apostolakis-2012	HAS-BLED vs. HEMORR_2_HAGES (+10.3%, *P* < 0.001); HAS-BLED vs. ATRIA(+13.0%, *P* < 0.001)	
Apostolakis-2013	Continuous: HAS-BLED vs. CHADS_2_ (+16.0%, *P* = 0.017); HAS-BLED vs. CHA_2_DS_2_-VASc (+29.0%, *P* < 0.001)	Not available
	Categorical: HAS-BLED vs. CHADS_2_ (+13.0%, *P* = 0.001); HAS-BLED vs. CHA_2_DS_2_-VASc (+10.0%, *P* = 0.04)	
Jaspers Focks-2016	HAS-BLED vs. HEMORR_2_HAGES (−5.61%, *P* = 0.194); HAS-BLED vs. ATRIA (−3.6%, *P* = 0.119)	Not available
Proietti-2018a	GARFIELD vs. HAS-BLED (+3.3%, *P* = 0.756);	GARFIELD vs. HAS-BLED (−0.1%, *P* = 0.746);
**Intracranial bleeding**		
Chao-2018	HAS-BLED vs. HEMORR_2_HAGES (+3.0%, *P* = 0.056); HAS-BLED vs. ATRIA (+6.0%, *P* < 0.001); HAS-BLED vs. ORBIT (+4.8%, *P* < 0.001)	Not available
Esteve-Pastor-2017	ABC vs. HAS-BLED (−13.96%, *P* = 0.075)	ABC vs. HAS-BLED (−0.11%, *P* = 0.536)
Yao-2017	HAS-BLED vs. CHA_2_DS_2_-VASc (+7.0%, *P* < 0.001)	Not available
**Gastrointestinal bleeding**		
Esteve-Pastor-2017	ABC vs. HAS-BLED (−8.17%, *P* = 0.362)	ABC vs. HAS-BLED (−5.55%, *P* = 0.164)
**Any bleeding**		
Jaspers Focks-2016	HAS-BLED vs. HEMORR_2_HAGES (−3.72%, *P* = 0.334); HAS-BLED vs. ATRIA (−8.51%, *P* = 0.009)	Not available
Proietti-2018a	GARFIELD vs. HAS-BLED (−8.7%, *P* < 0.001)	GARFIELD vs. HAS-BLED (−1.1%, *P* < 0.001)

*HAS-BLED, Hypertension, Abnormal liver/renal function, Stroke, Bleeding history or predisposition, Labile international normalized ratio, Elderly, Drugs/alcohol concomitantly; HEMORR_2_HAGES, Hepatic or renal disease, Ethanol abuse, Malignancy, Older, Reduced platelet count or function, Re-bleeding risk, Hypertension (uncontrolled), Anemia, Genetic factors, Excessive fall risk, Stroke; ATRIA, Anticoagulation and Risk Factors in Atrial Fibrillation; ORBIT, Outcomes Registry for Better Informed Treatment of Atrial Fibrillation; GARFIELD-AF, Global Anticoagulant Registry in the FIELD-Atrial Fibrillation; ABC, Age, Biomarkers, Clinical History; CHADS_2_, Congestive heart failure, Hypertension, Age ≥ 75 years, Diabetes mellitus, Stroke/transient ischemic attack history; CHA_2_DS_2_-VASc, Congestive heart failure/left ventricular ejection fraction ≤ 40%, Hypertension, Age ≥ 75 years, Diabetes mellitus, Stroke/transient ischemic attack/thromboembolism history, Vascular disease, Age 65–74 years, Sex (female); NRI, net reclassification improvement; IDI, integrated discrimination improvement; AF, atrial fibrillation*.

The NRI values of the ABC score compared with the HAS-BLED score had the reverse effects in two included studies [+13.8% in Berg et al. (26) and −13.74% in Esteve-Pastor et al. (27)]. In the study of Esteve-Pastor et al. (27), the ABC score showed significant negative IDI values compared with the HAS-BLED score (−13.14%, *p* = 0.002). Further study should confirm the improvement in the predictive accuracy of ABC vs. HAS-BLED scores in anticoagulated patients with AF.

The values from the NRI and IDI analyses assessing the improvement in predictive accuracy for any clinically relevant bleeding, any bleeding, intracranial bleeding, and gastrointestinal bleeding are presented in Table 3. The results of these sections should be interpreted cautiously due to the limiting number of included studies.

#### Calibration and Decision Curve Analysis

A total of seven included studies provided the calibration analysis of the HAS-BLED score, but the findings were inconsistent (Supplementary Table 5). Jaspers Focks et al. (24) and Beshir et al. (10) found that the HAS-BLED score had an adequate calibration. Two studies demonstrated that compared with the rates in the derivation cohort, the HAS-BLED score overestimated (28) or underestimated (29) the risk of bleeding. The HAS-BLED score had a better calibration than the ATRIA score (13, 30), but showed a similar or lower calibration compared with the ORBIT score (14, 30).

The net benefits of the HAS-BLED score vs. other risk scores were assessed by using the decision curve analysis (Supplementary Table 6). The HAS-BLED score might have larger net benefits than the HEMORR_2_HAGES, the CHADS_2_, the CHA_2_DS_2_-VASc, or the GARFIELD-AF scores (19, 25, 31). Two studies reported the net benefits between the HAS-BLED and the ABC scores, but reached the opposite conclusion (27, 32). The net benefits between the HAS-BLED and the ATRIA or the ORBIT scores might be related to the intervention thresholds (29).

## Discussion

In this study, our results suggested that the HAS-BLED score had moderate predictive abilities for bleeding risks in anticoagulated patients with AF regardless of the OAC type. We also observed the suitable application of the HAS-BLED score in patients with AF when the labile INR was unavailable. The discrimination performance of the HAS-BLED score assessed by the C-statistic was comparable to the HEMORR_2_HAGES, the ATRIA, the ORBIT, the GARFIELD-AF, or the ABC scores, but performed better than the CHADS_2_ or the CHA_2_DS_2_-VASc scores. The NRI and IDI data suggested that the HAS-BLED score performed better than the CHADS_2_ or the CHA_2_DS_2_-VASc scores and had similar or superior predictive ability compared with the HEMORR_2_HAGES, the ATRIA, the ORBIT, or the GARFIELD-AF scores. Calibration and decision curve analyses of the HAS-BLED score compared with other risk models required further evidence-based assessment due to the different findings among the included studies.

The use of OAC effectively reduces the embolic risks but at cost of an increased risk of bleeding. Over the past few decades, VKAs such as warfarin have been confirmed to be effective for preventing stroke in patients with AF. Since the effectiveness and safety of DOACs are superior or non-inferior to warfarin in patients with AF, DOACs are increasingly used with time. However, the bleeding events and their related cardiovascular outcomes are not negligible. The optimal use of VKAs or DOACs in the management of AF should be based on a balanced risk-to-benefit assessment during anticoagulation. For this situation, it is vital that the potentially preventable risk factors of bleeding events should be monitored sufficiently and addressed appropriately. The HAS-BLED score has been currently recommended by current AF guidelines, where a score of ≥3 points indicates high-risk bleeding. Note that the HAS-BLED score is previously derived and validated mainly in VKA-treated patients with AF, whether it could be used in DOAC-treated patients remains unclear. In this study, we found that the HAS-BLED score had moderate predictive values for bleeding risks in anticoagulated patients with AF and the findings were consistent in the subgroups of mixed anticoagulated drugs, DOACs, VKAs, and warfarin. The variable of labile INR in the HAS-BLED score was not available in all the included studies. Nevertheless, the predictive ability for major bleeding was not significantly changed, if we only included the studies with labile INR in the pooled analysis.

Although several other bleeding risk prediction models have been proposed in published studies, whether the predictive ability of these models is parallel to the guideline recommended HAS-BLED score remains unclear. The HAS-BLED score has been previously assessed as the most balanced bleeding risk prediction tool in terms of sensitivity and specificity by using a meta-analytic approach (5, 33). However, discrimination outcome data (sensitivity/specificity or the C-statistic) are less critical than other predictive accuracy outcome measures. In combination with NRI and IDI values, calibration, and decision curve analyses, our current meta-analysis comprehensively assessed the predictive abilities of the HAS-BLED vs. the HEMORR_2_HAGES, the ATRIA, the ORBIT, the GARFIELD-AF, or the ABC bleeding scores. Overall, as reflected by these multiple methods, the HAS-BLED score showed at least non-inferior abilities for bleeding risk prediction than the HEMORR_2_HAGES, the ATRIA, the ORBIT, or the GARFIELD-AF scores in VKA- or DOAC-treated patients with AF. Although there was no significant difference in the C-statistic between the HAS-BLED and the ABC scores, data of reclassification, calibration, and decision curve analyses between them were still controversial and needed further clarifications. In addition, there is an overlap of risk factors such as age, hypertension, previous stroke, and diabetes between stroke and bleeding risk scores. As such, the CHADS_2_ and the CHA_2_DS_2_-VASc stroke scores are also closely associated with the increased bleeding risks. Nevertheless, we found that the HAS-BLED score performed better than the CHADS_2_ or the CHA_2_DS_2_-VASc scores in anticoagulated patients with AF.

Several published studies have modified the HAS-BLED score by revising the original variables or including additional factors. As shown in Supplementary Table 7, integration of additional factors (e.g., biomarkers, gene polymorphisms, aortic stenosis, area deprivation index) on the basis of the original HAS-BLED score has been taken into account. The modified HAS-BLED score might improve the predictive ability, but certainly at the expense of additional complexity, increased cost, and reduced practicality. The number and definition of variables may vary from study to study, potentially affecting the diagnostic performance of the HAS-BLED score.

The dynamic changes of bleeding risks should be assessed in the management of AF (34). Chao et al. (35) found that the prediction values of the follow-up or the delta HAS-BLED score were better compared with the baseline HAS-BLED score. The HAS-BLED score has been tested prospectively in the mobile atrial fibrillation application-II (mAFA-II) randomized trial, suggesting that dynamic monitoring management could reduce major bleeding and increase OAC uptake at 1 year (36). Therefore, the dynamic changes in modifiable risk factors for bleeding outcomes during the follow-up should be monitored and corrected timely to improve AF patient care (36). Current evidence supports the HAS-BLED score regularly used in patients with AF to identify patients at high risk of bleeding as early as possible. However, the HAS-BLED score is sometimes inappropriately used in clinical practice as an excuse to preclude the use of oral anticoagulants. For the majority of patients with AF, the benefits of OAC in reducing the stroke risk outweigh the bleeding risk. Clinicians should flag up high bleeding-risk patients (e.g., the HAS-BLED score of ≥3) for the early review and follow-up. Appropriate monitoring services and more efforts are necessary to be taken to correct modifiable bleeding risk factors such as uncontrolled hypertension, poor control of INR (VKA users), concomitant use of medications such as aspirin or NSAIDs, and alcohol abuse.

The 2021 UK National Institute for Health and Care Excellence (NICE) guidelines tend to recommend the use of the ORBIT score in the bleeding risk prediction for patients with AF (especially for DOAC users) (37). This recommendation is mainly supported by the better calibration evidence than the HAS-BLED score although the low quality data. As the committee pointed out by using new risk models of the ORBIT score in current clinical practice, it remains a challenge potentially due to the unknown cost-effectiveness and extra resources. Clinicians are not familiar with the ORBIT score and learning and training may take time and cost. More importantly, the ORBIT bleeding score mainly consists of non-modifiable risk factors. In this context, a newly published study by Proietti et al. compared the abilities of the HAS-BLED vs. the ORBIT scores in contemporary patients with AF with DOACs based on the data from the European Society of Cardiology-European Heart Rhythm Association (ESC-EHRA) and the EURObservational Research Programme AF (EORP-AF) General Long-Term Registry (38). The authors found that the ORBIT score identified less patients at high bleeding risk, showed no improvements in predictive accuracy for major bleeding assessed by the NRI and IDI values, and had a poorer calibration compared with the HAS-BLED score (38). These findings seemingly do not support the use of the ORBIT over the HAS-BLED scores for bleeding risk prediction in patients with AF. The simple and practical use of the HAS-BLED score is still appropriate for assessing VKA- or DOAC- related bleeding risks and helps clinicians to make informed decisions in clinical practice.

### Limitations

There were still several limitations in this study. First, due to the high heterogeneity observed across the included studies, the discrimination performances of bleeding prediction models evaluated by the C-statistic should be interpreted cautiously. More studies focusing on the improvement in predictive accuracy by the NRI and IDI analyses, calibration, or net benefits by decision curve analysis could be taken to fully assess the performances of risk scores. Second, compared to the primary major bleeding, our results suggested a relatively lower predictive value of the HAS-BLED score for any clinically relevant bleeding, any bleeding, or intracranial bleeding. Only two studies reported the C-statistic for gastrointestinal bleeding. Therefore, more studies should further assess the value of the HAS-BLED score for these other bleeding outcomes. Third, the bleeding risk prediction tools of interest were derived and validated in studies with different study types ranging from observational cohorts to clinical trials, potentially complicating the synthesis of the C-statistic. Nevertheless, we observed no significant interaction in the subgroup analysis based on the study design. Fourth, we provided the data of concomitant antiplatelet drugs, but the effect of these drugs on the predictive value of the HAS-BLED score could not be analyzed due to the unclear cutoff points. Finally, this study was performed based on most included studies with low-quality data, but we comprehensively assessed the role of the HAS-BLED score vs. other risk models in predicting bleeding events in patients with AF, which had implications for clinical application and future research development.

## Conclusion

The HAS-BLED score had moderate predictive abilities for bleeding risks in VKA- or DOAC-treated patients with AF. The HAS-BLED score was at least non-inferior to the HEMORR_2_HAGES, the ATRIA, the ORBIT, the GARFIELD-AF, or the ABC scores, but performed better than the CHADS_2_ or the CHA_2_DS_2_-VASc scores.

## Data Availability Statement

The original contributions presented in the study are included in the article/Supplementary Material, further inquiries can be directed to the corresponding authors.

## Author Contributions

All authors listed have made a substantial, direct, and intellectual contribution to the work and approved it for publication.

## Funding

This study was funded by the National Natural Science Foundation of China (82100273), the China Postdoctoral Science Foundation (2020M673016), and the China National Postdoctoral Program for Innovative Talents (BX20200400).

## Conflict of Interest

The authors declare that the research was conducted in the absence of any commercial or financial relationships that could be construed as a potential conflict of interest.

## Publisher's Note

All claims expressed in this article are solely those of the authors and do not necessarily represent those of their affiliated organizations, or those of the publisher, the editors and the reviewers. Any product that may be evaluated in this article, or claim that may be made by its manufacturer, is not guaranteed or endorsed by the publisher.
