# Supplementary material for: Diagnostic Accuracy of the HAS-BLED Bleeding Score in VKA- or DOAC-Treated Patients With Atrial Fibrillation: A Systematic Review and Meta-Analysis
Source: Front Cardiovasc Med. 2021 Nov 22;8:757087. doi: 10.3389/fcvm.2021.757087 (PMC8648046; doi:10.3389/fcvm.2021.757087)
Supplement: Supplementary file 1 [file Data_Sheet_1.PDF]

Supplemental Table 1. The algorithms for the bleeding risk scores

Models	Variables	Score
HAS-BLED	Uncontrolled hypertension (systolic blood pressure >160 mmHg)	1
	Impaired renal function (dialysis, transplant, Cr >2.26 mg/dL or >200 µmol/L) or impaired liver function (cirrhosis or bilirubin >2x normal or AST/ALT/AP >3x normal)	1 or 2
	History of stroke	1
	Bleeding history or predisposition (anemia)	1
	Labile international normalized ratio (INR) (< 60% of time in therapeutic range)	1
	Elderly (>65 years)	1
	Concomitant use of antiplatelet agents, nonsteroidal antiinflammatory drugs, and alcohol consumption (more than 8 units/week)	1 or 2
ATRIA	Anemia	3
	Severe renal impairment (GFR <30 mL/min or dialysis-dependent)	3
	Age ≥75 years	2
	Any prior hemorrhage diagnosis	1
	Hypertension history	1
ORBIT	Age ≥75 years	1
	Reduced haemoglobin/haematocrit/history of anaemia	2
	History of bleeding	2
	Impaired renal function (<60 mL/min/1.73 m^2)	1
	Treatment with antiplatelets	1
$HEMORR_2HAGES$	Hepatic or renal disease	1
	Alcohol abuse	1
	Malignancy history	1
	Age >75 years	1
	Reduced platelet count or function	1
	History of bleeding	2

	Uncontrolled hypertension	1
	Anemia	1
	Genetic factors (CYP 2C9 single-nucleotide polymorphisms)	1
	Excessive fall risk	1
	History of stroke	1
$CHADS_2$	Congestive heart failure	1
	Hypertension	1
	Age ≥75 years	1
	Diabetes mellitus	1
	Stroke/transient ischemic attack history	2
CHA_2DS_2-VASc	Congestive heart failure/left ventricular ejection fraction ≤ 40%	1
	Hypertension	1
	Age ≥75 years	2
	Diabetes mellitus	1
	Stroke/transient ischemic attack/thromboembolism history	2
	Vascular disease	1
	Age 65–74 years	1
	Sex (female)	1
ABC bleeding score	Age	-
	Biomarkers (hematocrit, high sensitivity troponin T (hsTnT), GDF-15)	-
	Clinical history (prior bleeding)	-
GARFIELD-AF	Age, pulse, moderate to severe chronic kidney disease (class III-V), history of vascular disease, history of bleeding, Carotid occlusive disease , Diabetes (type 1/2), Current use of antiplatelet drugs (e.g. aspirin, clopidogrel)	-

Abbreviations: HAS-BLED=Hypertension, Abnormal liver/renal function, Stroke, Bleeding history or predisposition, Labile international normalized ratio, Elderly, Drugs/alcohol concomitantly; $HEMORR_2HAGES$ =Hepatic or renal disease, Ethanol abuse, Malignancy, Older, Reduced platelet count or function, Re-bleeding risk, Hypertension (uncontrolled), Anemia, Genetic factors, Excessive fall risk, Stroke; ATRIA=Anticoagulation and Risk Factors in Atrial Fibrillation; ORBIT=Outcomes Registry for Better Informed Treatment of Atrial Fibrillation; GARFIELD-AF=Global Anticoagulant Registry in the FIELD–Atrial Fibrillation; ABC=Age, Biomarkers, Clinical History; $CHADS_2$=Congestive heart failure, Hypertension, Age ≥75 years, Diabetes mellitus, Stroke/transient

ischemic attack history; CHA_2DS_2-VASc=Congestive heart failure/left ventricular ejection fraction ≤ 40%, Hypertension, Age ≥ 75 years, Diabetes mellitus, Stroke/transient ischemic attack/thromboembolism history, Vascular disease, Age 65–74 years, Sex (female).

Supplemental Table 2. Included studies and excluded studies of this meta-analysis

Included studies

[1]. Pisters, R., et al., A novel user-friendly score (HAS-BLED) to assess 1-year risk of major bleeding in patients with atrial fibrillation: the Euro Heart Survey. Chest, 2010. 138(5): p. 1093-100.
[2]. Olesen, J.B., et al., Bleeding risk in 'real world' patients with atrial fibrillation: comparison of two established bleeding prediction schemes in a nationwide cohort. J Thromb Haemost, 2011. 9(8): p. 1460-7.
[3]. Apostolakis, S., et al., Performance of the HEMORR(2)HAGES, ATRIA, and HAS-BLED bleeding risk-prediction scores in patients with atrial fibrillation undergoing anticoagulation: the AMADEUS (evaluating the use of SR34006 compared to warfarin or acenocoumarol in patients with atrial fibrillation) study. J Am Coll Cardiol, 2012. 60(9): p. 861-7.
[4]. Apostolakis, S., et al., Comparison of the CHADS2, CHA2DS2-VASc and HAS-BLED scores for the prediction of clinically relevant bleeding in anticoagulated patients with atrial fibrillation: the AMADEUS trial. Thromb Haemost, 2013. 110(5): p. 1074-9.
[5]. Senoo, K., et al., Evaluation of the HAS-BLED, ATRIA, and ORBIT Bleeding Risk Scores in Patients with Atrial Fibrillation Taking Warfarin. The American Journal of Medicine, 2016. 129(6): p. 600-607.
[6]. Proietti, M., et al., Major Bleeding in Patients with Non-Valvular Atrial Fibrillation: Impact of Time in Therapeutic Range on Contemporary Bleeding Risk Scores. Scientific Reports, 2016. 6: p. 24376.
[7]. Proietti, M., et al., Predicting Bleeding Events in Anticoagulated Patients With Atrial Fibrillation: A Comparison Between the HAS‐BLED and GARFIELD‐AF Bleeding Scores. Journal of the American Heart Association, 2018. 7(18).
[8]. Proietti, M., et al., Comparison of bleeding risk scores in patients with atrial fibrillation: insights from the RE-LY trial. J Intern Med, 2018. 283(3): p. 282-292.
[9]. Friberg, L., M. Rosenqvist and G.Y. Lip, Evaluation of risk stratification schemes for ischaemic stroke and bleeding in 182 678 patients with atrial fibrillation: the Swedish Atrial Fibrillation cohort study. Eur Heart J, 2012. 33(12): p. 1500-10.
[10]. Roldan, V., et al., Predictive value of the HAS-BLED and ATRIA bleeding scores for the risk of serious bleeding in a "real-world" population with atrial fibrillation receiving anticoagulant therapy. Chest, 2013. 143(1): p. 179-84.
[11]. Roldan, V., et al., The HAS-BLED score has better prediction accuracy for major bleeding than CHADS2 or CHA2DS2-VASc scores in anticoagulated patients with atrial fibrillation. J Am Coll Cardiol, 2013. 62(23): p. 2199-204.
[12]. Hijazi, Z., et al., The novel biomarker-based ABC (age, biomarkers, clinical history)-bleeding risk score for patients with atrial fibrillation: a derivation and validation study. The Lancet, 2016. 387(10035): p. 2302-2311.
[13]. Esteve-Pastor, M.A., et al., Is the ORBIT Bleeding Risk Score Superior to the HAS-BLED Score in Anticoagulated Atrial Fibrillation Patients? Circulation Journal, 2016. 80(10): p. 2102-2108.
[14]. Jaspers Focks, J., et al., Low performance of bleeding risk models in the very elderly with atrial fibrillation using vitamin K antagonists. Journal of Thrombosis and Haemostasis, 2016. 14(9): p. 1715-1724.
[15]. Berg, D.D., et al., Performance of the ABC Scores for Assessing the Risk of Stroke or Systemic Embolism and Bleeding in Patients With Atrial Fibrillation in ENGAGE AF-TIMI 48. Circulation, 2019. 139(6): p. 760-771.
[16]. Steinberg, B.A., et al., How well does physician risk assessment predict stroke and bleeding in atrial fibrillation? Results from the Outcomes Registry for Better Informed Treatment of Atrial Fibrillation (ORBIT-AF). American Heart Journal, 2016. 181: p. 145-152.
[17]. Rivera-Caravaca, J.M., et al., Importance of time in therapeutic range on bleeding risk prediction using clinical risk scores in patients with atrial fibrillation. Scientific Reports, 2017. 7(1).
[18]. Esteve-Pastor, M.A., et al., Long-term bleeding risk prediction in 'real world' patients with atrial fibrillation: Comparison of the HAS-BLED and ABC-Bleeding risk scores. The Murcia Atrial Fibrillation Project. Thromb Haemost, 2017. 117(10): p. 1848-1858.
[19]. Caro Martínez, C., et al., Comparison of Bleeding Risk Scores in Patients With Nonvalvular Atrial Fibrillation Starting Direct Oral Anticoagulants. Revista Española de Cardiología (English Edition), 2017. 70(10): p. 878-880.
[20]. Poli, D., et al., Comparison of HAS-BLED and HAS-BED Versus CHADS 2 and CHA 2 DS 2 VASC Stroke and Bleeding Scores in Patients With Atrial Fibrillation. The American Journal of Cardiology, 2017. 119(7): p. 1012-1016.
[21]. Chao, T., et al., Major bleeding and intracranial hemorrhage risk prediction in patients with atrial fibrillation: Attention to modifiable bleeding risk factors or use of a bleeding risk stratification score? A nationwide cohort study. International Journal of Cardiology, 2018. 254: p. 157-161.
[22]. Beshir, S.A., et al., Evaluation of the predictive performance of bleeding risk scores in patients with non-valvular atrial fibrillation on oral anticoagulants. Journal of Clinical Pharmacy and Therapeutics, 2018. 43(2): p. 209-219.
[23]. Fox, K.A.A., et al., GARFIELD-AF risk score for mortality, stroke, and bleeding within 2 years in patients with atrial fibrillation. European Heart Journal - Quality of Care and Clinical Outcomes, 2021.
[24]. Dalgaard, F., et al., GARFIELD-AF model for prediction of stroke and major bleeding in atrial fibrillation: a Danish nationwide validation study. BMJ Open, 2019. 9(11): p. e033283.
[25]. Mori, N., et al., External Validation of the ORBIT Bleeding Score and the HAS-BLED Score in Nonvalvular Atrial Fibrillation Patients Using Direct Oral Anticoagulants (Asian Data from the DIRECT Registry). The American Journal of Cardiology, 2019. 124(7): p. 1044-1048.
[26]. O'Brien, E.C., et al., The ORBIT bleeding score: a simple bedside score to assess bleeding risk in atrial fibrillation. Eur Heart J, 2015. 36(46): p. 3258-64.
[27]. Quinn, G.R., et al., How Well Do Stroke Risk Scores Predict Hemorrhage in Patients With Atrial Fibrillation? The American Journal of Cardiology, 2016. 118(5): p. 697-699.

[28]. Elvira-Ruiz, G., et al., Aortic valve stenosis provides complementary information to bleeding risk scores in non-valvular atrial fibrillation patients initiating anticoagulation. J Geriatr Cardiol, 2020. 17(3): p. 141-148.
[29]. Claxton, J.N.S., et al., A new model to predict major bleeding in patients with atrial fibrillation using warfarin or direct oral anticoagulants. PLOS ONE, 2018. 13(9): p. e0203599.
[30]. Rutherford, O.W., et al., New score for assessing bleeding risk in patients with atrial fibrillation treated with NOACs. Open Heart, 2018. 5(2): p. e000931.
[31]. Yao, X., et al., Comparison of the CHA2DS2-VASc, CHADS2, HAS-BLED, ORBIT, and ATRIA Risk Scores in Predicting Non-Vitamin K Antagonist Oral Anticoagulants-Associated Bleeding in Patients With Atrial Fibrillation. Am J Cardiol, 2017. 120(9): p. 1549-1556.
[32]. Adam, L., et al., Novel bleeding risk score for patients with atrial fibrillation on oral anticoagulants, including direct oral anticoagulants. Journal of Thrombosis and Haemostasis, 2021. 19(4): p. 931-940.
[33]. Prochaska, J.H., et al., Sustained atrial fibrillation increases the risk of anticoagulation-related bleeding in heart failure. Clinical Research in Cardiology, 2018. 107(12): p. 1170-1179.
[34]. Siu, C., et al., Risk of stroke and intracranial hemorrhage in 9727 Chinese with atrial fibrillation in Hong Kong. Heart Rhythm, 2014. 11(8): p. 1401-1408.
[35]. Suzuki, M., et al., Improvement of HAS-BLED bleeding score predictive capability by changing the definition of renal dysfunction in Japanese atrial fibrillation patients on anticoagulation therapy. Journal of Cardiology, 2014. 64(6): p. 482-487.
[36]. Ravvaz, K., et al., Evaluating the effects of socioeconomic status on stroke and bleeding risk scores and clinical events in patients on oral anticoagulant for new onset atrial fibrillation. PLOS ONE, 2021. 16(3): p. e0248134.
[37]. Schwartz, S.M., et al., Discriminative Ability of CHA2DS2-VASc and HAS-BLED Score in Whites and Nonwhites. The American Journal of Cardiology, 2019. 123(12): p. 1949-1954.
[38]. Barnes, G.D., et *al., The predicti*ve ability of the CHADS2 and CHA2DS2-VASc scores for bleeding risk in atrial fibrillation: the MAQI(2) experience. Thromb Res, 2014. 134(2): p. 294-9.
[39]. Lip, G.Y.H., et al., The HAS-BLED, ATRIA, and ORBIT Bleeding Scores in Atrial Fibrillation Patients Using Non-Vitamin K Antagonist Oral Anticoagulants. The American Journal of Medicine, 2018. 131(5): p. 574.e13-574.e27.

Excluded studies

Criteria 1. Patients with OACs were not analyzed separately.

[1]. Herrin, J., et al., Comparative Effectiveness of Machine Learning Approaches for Predicting Gastrointestinal Bleeds in Patients Receiving Antithrombotic Treatment. JAMA Network Open, 2021. 4(5): p. e2110703.
[2]. Morrone, D., et al., Mortality Prediction of the CHA2DS2-VASc Score, the HAS-BLED Score, and Their Combination in Anticoagulated Patients with Atrial Fibrillation. Journal of Clinical Medicine, 2020. 9(12): p. 3987.
[3]. Jaakkola, S., et al., Usefulness of the CHA2DS2-VASc and HAS-BLED Scores in Predicting the Risk of Stroke Versus Intracranial Bleeding in Patients With Atrial Fibrillation (from the FibStroke Study). Am J Cardiol, 2018. 121(10): p. 1182-1186.
[4]. Lip, G.Y., et al., Assessing the risk of bleeding in patients with atrial fibrillation: the Loire Valley Atrial Fibrillation project. Circ Arrhythm Electrophysiol, 2012. 5(5): p. 941-8.
[5]. Guo Y., et al., Assessing bleeding risk in 4824 Asian patients with atrial fibrillation: The Beijing PLA Hospital Atrial Fibrillation Project. SCI REP-UK. 2016;6:31755.

Criteria 2. Duplicate data with the included studies

[1]. Hijazi Z,et al. Growth-differentiation factor 15 and risk of major bleeding in atrial fibrillation: Insights from the Randomized Evaluation of Long-Term Anticoagulation Therapy (RE-LY) trial. AM HEART J. 2017;190:94-103. (1.overlapping with Hijazi-2016; data source: The RE-LY trial; 2. compared with Hijazi-2016, Hijazi-2017 mainly aimed to study the modified HAS-BLED version by adding growth-differentiation factor 15 into the original HAS-BLED score)
[2]. Fox, K.A.A., et al., Improved risk stratification of patients with atrial fibrillation: an integrated GARFIELD-AF tool for the prediction of mortality, stroke and bleed in patients with and without anticoagulation. BMJ Open, 2017. 7(12): p. e017157. (1.overlapping with Fox-2021, data source: The GARFIELD-AF registry; 2. compared with Fox-2017, the included study of Fox-2021 was newly published and had higher sample size)
[3]. García-Fernández, A., et al., Does von Willebrand factor improve the predictive ability of current risk stratification scores in patients with atrial fibrillation? Scientific Reports, 2017. 7(1). (1.overlapping with Rivera-Caravaca-2017, data source: Single anticoagulation centre in a tertiary hospital in Murcia, Spain; 2. compared with Rivera-Caravaca-2017, García-Fernández-2017 mainly aimed to study the modified HAS-BLED version by adding von Willebrand factor into the original HAS-BLED score)
[4]. Serna, M.J., et al., Pharmacogenetics of vitamin K antagonists and bleeding risk prediction in atrial fibrillation. European Journal of Clinical Investigation, 2018. 48(6): p. e12929. (1.overlapping with Rivera-Caravaca-2017, data source: Single anticoagulation centre in a tertiary hospital in Murcia, Spain; 2. compared with Rivera-Caravaca-2017, Serna-2018 mainly aimed to study the modified HAS-BLED version by adding gene polymorphisms into the original HAS-BLED score)
[5]. Roldán V, et al. Enhancing the 'real world' prediction of cardiovascular events and major bleeding with the CHA2DS2-VASc and HAS-BLED scores using multiple biomarkers. Annals of medicine (Helsinki). 2018;50(1):26-34. (1.overlapping with Rivera-Caravaca-2017, data source: Single anticoagulation centre in a tertiary hospital in Murcia, Spain; 2. compared with Rivera-Caravaca-2017, Roldán-2018 mainly aimed to study the modified HAS-BLED version by adding biomarkers into the original HAS-BLED score)
[6]. Chao, T., et al., Incident Risk Factors and Major Bleeding in Patients with Atrial Fibrillation Treated with Oral Anticoagulants: A Comparison of Baseline, Follow-up and Delta HAS-BLED Scores with an Approach Focused on Modifiable Bleeding Risk Factors. Thrombosis and Haemostasis, 2018. 47(04): p. 768-777. (1.overlapping with Chao-2018[INT J CARDIOL], data source: National Health Insurance Research Database, Taiwan; 2. compared with Chao-2018[INT J CARDIOL], this study aimed to study the HAS-BLED and dynamic bleeding risk)
[7]. Wang SV, et al. Prediction of rates of thromboembolic and major bleeding outcomes with dabigatran or warfarin among patients with atrial fibrillation: new initiator cohort study. BMJ. 2016:i2607. (overlapping with

Yao-2017 and Claxton-2018)
[8]. Gallego, P., et al., Relation of the HAS-BLED bleeding risk score to major bleeding, cardiovascular events, and mortality in anticoagulated patients with atrial fibrillation. Circ Arrhythm Electrophysiol, 2012. 5(2): p. 312-8. (overlapping with Roldan-2013)
[9]. Rivera-Caravaca JM, Marín F, Vilchez JA, Gálvez J, Esteve-Pastor MA, Vicente V, Lip GYH, Roldán V. Refining Stroke and Bleeding Prediction in Atrial Fibrillation by Adding Consecutive Biomarkers to Clinical Risk Scores. STROKE. 2019;50(6):1372-1379. (1.overlapping with Rivera-Caravaca-2017, data source: Single anticoagulation centre in a tertiary hospital in Murcia, Spain; 2. compared with Rivera-Caravaca-2017, this study mainly aimed to study the modified HAS-BLED version by adding vWF and biomarkers into the original HAS-BLED score)

Criteria 3. Anticoagulated drugs were not VKAs or DOACs, or unknown OAC information, or non-anticoagulated population
[1]. Esteve-Pastor, M., et al., Assessing Bleeding Risk in Atrial Fibrillation Patients: Comparing a Bleeding Risk Score Based Only on Modifiable Bleeding Risk Factors against the HAS-BLED Score. The AMADEUS Trial. Thrombosis and Haemostasis, 2017. 117(12): p. 2261-2266.
[2].Senoo, K. and G.Y. Lip, Predictive abilities of the HAS-BLED and ORBIT bleeding risk scores in non-warfarin anticoagulated atrial fibrillation patients: An ancillary analysis from the AMADEUS trial. Int J Cardiol, 2016. 221: p. 379-82.
[3].Fauchier, L., et al., Predictive ability of HAS-BLED, HEMORR2HAGES, and ATRIA bleeding risk scores in patients with atrial fibrillation. A French nationwide cross-sectional study. International Journal of Cardiology, 2016. 217: p. 85-91.
[4].Nielsen, P.B., T.B. Larsen and G. Lip, Recalibration of the HAS-BLED Score: Should Hemorrhagic Stroke Account for One or Two Points? Chest, 2016. 149(2): p. 311-314.
[5].Apostolakis S, Lane DA, Guo Y, Buller H, Lip GY. Performance of the HEMORR 2 HAGES, ATRIA, and HAS-BLED bleeding risk-prediction scores in nonwarfarin anticoagulated atrial fibrillation patients. J AM COLL CARDIOL. 2013;61(3):386-387.
[6].Lip, G.Y., et al., Comparative validation of a novel risk score for predicting bleeding risk in anticoagulated patients with atrial fibrillation: the HAS-BLED (Hypertension, Abnormal Renal/Liver Function, Stroke, Bleeding History or Predisposition, Labile INR, Elderly, Drugs/Alcohol Concomitantly) score. J Am Coll Cardiol, 2011. 57(2): p. 173-80.
[7]Guo Y., et al., Validation of contemporary stroke and bleeding risk stratification scores in non-anticoagulated Chinese patients with atrial fibrillation. INT J CARDIOL. 2013;168(2):904-909.
[8]Guo Y, et al., Comparing Bleeding Risk Assessment Focused on Modifiable Risk Factors Only Versus Validated Bleeding Risk Scores in Atrial Fibrillation. AM J MED. 2018;131(2):185-192.

Criteria 4. No data of C-index, or CIs of the C-index
[1]. Gorman, E.W., et al., Validation Of The HAS-BLED Tool In Atrial Fibrillation Patients Receiving Rivaroxaban. Journal of atrial fibrillation, 2016. 9(2): p. 1461.
[2]. Al-Turaiki, A., et al., Assessment and comparison of CHADS2, CHA2DS2-VASc, and HAS-BLED scores in patients with atrial fibrillation in Saudi Arabia. Annals of Thoracic Medicine, 2016. 11(2): p. 146.
[3]. Peacock, W.F., et al., CHA2DS2-VASc Scores and Major Bleeding in Patients With Nonvalvular Atrial Fibrillation Who Are Receiving Rivaroxaban. Ann Emerg Med, 2016.
[4]. Blann, A.D. and G.Y. Lip, Renal, endothelial function, warfarin management, and the CHADS2, CHA2DS2VASc and HAS-BLED scores inpredicting MACE in AF. Thromb Haemost, 2015. 113(5): p. 1155-7.
[5]. Abumuaileq, R.R., et al., Comparative evaluation of HAS-BLED and ATRIA scores by investigating the full potential of their bleeding prediction schemes in non-valvular atrial fibrillation patients on vitamin-K antagonists. Int J Cardiol, 2014. 176(3): p. 1259-61.
[6]. Hijazi, Z., et al., High-Sensitivity Troponin I for Risk Assessment in Patients With Atrial Fibrillation. Circulation, 2014. 129(6): p. 625-634.
[7]. Naganuma M., et al., Clinical outcome in Japanese elderly patients with non-valvular atrial fibrillation taking warfarin: a single-center observational study. THROMB RES. 2012;130(1):21-26.
[8]. Guo Y., et al., Regular Bleeding Risk Assessment Associated with Reduction in Bleeding Outcomes: The mAFA-II Randomized Trial. The American Journal of Medicine. 2020;133(10):1195-1202.

Criteria 5. non-AF population or AF patients with specific disease
[1]. Frei, A.N., et al., Comparison of Bleeding Risk Scores in Elderly Patients Receiving Extended Anticoagulation with Vitamin K Antagonists for Venous Thromboembolism. Thromb Haemost, 2021.
[2]. Zhang, Q., et al., Relationship of the ORBIT and HAS-BLED scores with Killip class 3-4 in patients with ST-segment elevation myocardial infarction. Medicine, 2019. 98(8): p. e14578.
[3]. Yoshida, R., et al., Performance of HAS-BLED, ORBIT, PRECISE-DAPT, and PARIS risk score for predicting long-term bleeding events in patients taking an oral anticoagulant undergoing percutaneous coronary intervention. Journal of Cardiology, 2018.
[4]. Hilkens, N.A., A. Algra and J.P. Greving, Predicting Major Bleeding in Ischemic Stroke Patients With Atrial Fibrillation. Stroke, 2017. 48(11): p. 3142-3144.
[5]. Mueller, K., et al., HAS-BLED Predicts Warfarin Control in Australian Patients treated for Deep Vein Thrombosis. Basic & Clinical Pharmacology & Toxicology, 2017. 120(3): p. 299-302.
[6]. Hsieh, M., et al., Predictive performance of HAS-BLED risk score for long-term survival in patients with non-ST elevated myocardial infarction without atrial fibrillation. Journal of Cardiology, 2017. 69(1): p. 136-143.
[7]. Kemal, H.S., et al., Utility of CHA2DS2-VASc and HAS-BLED Scores as Predictor of Thromboembolism and Bleeding After Left Ventricular Assist Device Implantation. ASAIO Journal, 2017. 63(6): p. 720-724.
[8]. Shah, R.R., et al., Utility of the HAS-BLED Score in Risk Stratifying Patients on Dual Antiplatelet Therapy Post 12 Months After Drug-Eluting Stent Placement. Catheterization and Cardiovascular Interventions, 2017. 89(4): p. E99-E103.
[9]. Chatterjee, S., G.Y. Lip and J. Giri, HAS-BLED Versus ATRIA Risk Scores for Intracranial Hemorrhage in Patients Receiving Thrombolytics for Pulmonary Embolism. J Am Coll Cardiol, 2016. 67(24): p. 2904-5.

[10]. Kataoka, T., K. Hoshi and T. Ando, Is the HAS-BLED score useful in predicting post-extraction bleeding in patients taking warfarin? A retrospective cohort study. BMJ Open, 2016. 6(3): p. e010471.
[11]. Liu, J., et al., Predicting the Outcomes of Acute Ischemic Stroke with Rheumatic Heart Disease: The Values of CHADS2, CHA2DS2–VASc, and HAS-BLED Scores. Journal of Stroke and Cerebrovascular Diseases, 2016. 25(3): p. 722-726.
[12]. Desai, A.K., et al., Utility of Inferior Vena Cava Filters in Severe Pulmonary Embolism, Catheter-directed Therapy in Massive and Submassive Pulmonary Embolism, and HAS-BLED Score to Determine Risk of Major Hemorrhage in Pulmonary Embolism. American Journal of Respiratory and Critical Care Medicine, 2016. 193(11): p. 1301-1303.
[13]. Hsieh, M., et al., HAS-BLED score predicts risk of in-hospital major bleeding in patients with acute non-ST segment elevation myocardial infarction. Thrombosis Research, 2015. 136(4): p. 775-780.
[14]. Konishi, H., et al., Impact of the HAS-BLED Score on Long-Term Outcomes After Percutaneous Coronary Intervention. The American Journal of Cardiology, 2015. 116(4): p. 527-531.
[15]. Capodanno, D., et al., Predictive accuracy of CHA2DS2-VASc and HAS-BLED scores in patients without atrial fibrillation undergoing percutaneous coronary intervention and discharged on dual antiplatelet therapy. International Journal of Cardiology, 2015. 199: p. 319-325.
[16]. Kooiman, J., et al., The HAS-BLED Score Identifies Patients with Acute Venous Thromboembolism at High Risk of Major Bleeding Complications during the First Six Months of Anticoagulant Treatment. PLOS ONE, 2015. 10(4): p. e0122520.
[17]. Seet, R.C., et al., Bleeding complications associated with warfarin treatment in ischemic stroke patients with atrial fibrillation: a population-based cohort study. J Stroke Cerebrovasc Dis, 2013. 22(4): p. 561-9.
[18]. Lip, G.Y.H., et al., Comparative assessment of the HAS-BLED score with other published bleeding risk scoring schemes, for intracranial haemorrhage risk in a non-atrial fibrillation population: The Chin-Shan Community Cohort Study. International Journal of Cardiology, 2013. 168(3): p. 1832-1836.
[19].Tchen S., et al., Validation of Bleeding Risk Prediction Scores for Patients With Major Bleeding on Direct Oral Anticoagulants. ANN PHARMACOTHER. 2020;54(12):1175-1184.
[20].Wang TKM., et al., Relationships between Anticoagulation, Risk Scores and Adverse Outcomes in Dialysis Patients with Atrial Fibrillation. Heart, Lung and Circulation. 2016;25(3):243-249.
[21].Ocak G., et al., Performance of bleeding risk scores in dialysis patients. NEPHROL DIAL TRANSPL. 2019;34(7):1223-1231.
[22].Puurunen MK., et al., CHADS2, CHA2DS2-VASc and HAS-BLED as predictors of outcome in patients with atrial fibrillation undergoing percutaneous coronary intervention. THROMB RES. 2014;133(4):560-566.
[23].Lip GYH, Jensen M, Melgaard L, Skjoth F, Nielsen PB, Larsen TB. Stroke and bleeding risk scores in patients with atrial fibrillation and valvular heart disease: evaluating 'valvular heart disease' in a nationwide cohort study. EUROPACE. 2019;21(1):33-40.
[24].Benito-Gonzalez, T., et al., Incidence and prognostic implications of late bleeding events after percutaneous mitral valve repair. Int J Cardiol Heart Vasc, 2018. 21: p. 16-21.
[25].Donzé, J., et al., Scores to Predict Major Bleeding Risk During Oral Anticoagulation Therapy: A Prospective Validation Study. The American Journal of Medicine, 2012. 125(11): p. 1095-1102.

Criteria 6. the risk of bleeding was not analyzed separately.

[1].Lip G, et al., Development of a novel composite stroke and bleeding risk score in patients with atrial fibrillation: the AMADEUS Study. CHEST. 2013;144(6):1839-1847.
[2].Banerjee A, et al., Composite risk scores and composite endpoints in the risk prediction of outcomes in anticoagulated patients with atrial fibrillation. The Loire Valley Atrial Fibrillation Project. Thromb Haemost. 2014;111(3):549-556.
[3].Rivera-Caravaca, J.M., et al., Prediction of long-term net clinical outcomes using the TIMI-AF score: Comparison with CHA2DS2-VASc and HAS-BLED. The American heart journal, 2018. 197: p. 27-34.

Supplemental Table 3. Variables of the HAS-BLED score in the included studies

	**H: Hypertension (1 point)**	**A: Abnormal renal and liver function (1 point each)**	**S: Stroke (1 point)**	**B: Bleeding (1 point)**	**L: Labile INRs (1 point)**	**E: Elderly (>65 years) (1 point)**	**D: Drugs or alcohol (1 point each)**
Pisters -2010	√	√	√	√	√	√	√
Olesen-2011	√	√	√	√	Unavailable	√	√
Proietti-2016	√	Abnormal liver function：Unavailable	√	√	√	√	√
Proietti-2018a	√	Abnormal liver function：Unavailable	√	√	√	√	√
Apostolakis-2012; Apostolakis-2013; Senoo-2016	√	√	√	Unavailable	Each patient' first five INR measurements following study entry to calculate TTR	√	Drug abuse: Unavailable
Friberg-2012	√	√	√	√	Unavailable	√	NSAID: Unavailable
Roldan-2013a Roldan-2013b	√	√	√	√	Unavailable	√	√
Barnes-2014	√	√	√	√	Unavailable	√	√
Esteve-Pastor-2016	√	√	√	√	√*	√	√
Hijazi-2016	√	√	√	√	√*	√	√
Proietti-2018b	√	√	√	√	√*	√	√
Berg-2019	√	√	√	√	√*	√	√
Jaspers Focks-2016	√	√	√	√	√	√	√
Steinberg-2016	√	√	√	√	Unavailable	√	√
Poli-2017	√	√	√	√	√	√	√

Caro Martínez-2017	Unspecified	Unspecified	Unspecified	Unspecified	not applicable	Unspecified	Unspecified
Esteve-Pastor-2017; Rivera-Caravaca-2017	√	√	√	√	√	√	√
Fox-2021	√	√	√	√	Unavailable	√	√
Beshir-2018	√	√	√	√	Unavailable	√	√
Chao-2018	√	√	√	√	Unavailable	√	√
Dalgaard-2019	√	√	√	√	Unavailable	√	√
Lip-2018	√	√	√	√	Unavailable	√	√
Mori-2019	√	√	√	√	not applicable	√	√
O'Brien-2015	√	√	√	√	Unavailable	√	√
Quinn-2016	√	√	√	√	√	√	Aspirin or NSAID: Unavailable
Yao-2017	√	√	√	√	not applicable	√	√
Claxton-2018	√	√	√	√	Unavailable	√	√
Elvira-Ruiz-2020	√	√	√	√	Unavailable	√	√
Rutherford-2018	√	liver enzymes: Unavailable	√	√	Unavailable	√	drug abuse: Unavailable
Adam-2021	√	√	√	√	√	√	√
Siu-2014	Unspecified	Unspecified	Unspecified	Unspecified	Unspecified	Unspecified	Unspecified
Suzuki-2014	√	√	√	√	√	√	√
Prochaska-2018	Unspecified	Unspecified	Unspecified	Unspecified	Unspecified	Unspecified	Unspecified
Schwartz-2019	√	prior stroke: Unavailable	√	√	Unavailable	√	√
Ravvaz-2021	√	√	√	√	√	√	√

[*] only applies to a VKA user, not applicable for a non-VKA user

Abbreviations: HAS-BLED=Hypertension, Abnormal liver/renal function, Stroke, Bleeding history or predisposition, Labile international normalized ratio, Elderly, Drugs/alcohol concomitantly; INR=international normalized ratio.

Supplemental Table 4. The risk of bias assessment for all included studies using the PROBAST checklist

	Risk of Bias				Overall
	Participants	Predictors	Outcomes	Analysis	
Pisters -2010	Low risk	Unclear	High risk	Unclear	High risk
Olesen-2011	Low risk	Low risk	High risk	Low risk	High risk
Proietti-2016	Low risk	Unclear	Unclear	Unclear	Unclear
Proietti-2018a	Low risk	Unclear	Low risk	Unclear	Unclear
Apostolakis-2012	Low risk	Unclear	High risk	Low risk	High risk
Apostolakis-2013	Low risk	Unclear	High risk	Low risk	High risk
Senoo-2016	Low risk	Unclear	High risk	Unclear	High risk
Friberg-2012	Low risk	Unclear	Unclear	Unclear	Unclear
Roldan-2013a	Low risk	Unclear	High risk	Unclear	High risk
Roldan-2013b	Low risk	Low risk	Unclear	Unclear	Unclear
Barnes-2014	Low risk	Unclear	High risk	Unclear	High risk
Esteve-Pastor-2016	Low risk	Unclear	High risk	Unclear	High risk
Hijazi-2016	Low risk	Unclear	Unclear	Unclear	Unclear
Proietti-2018b	Low risk	Unclear	Low risk	Unclear	Unclear
Berg-2019	Low risk	Unclear	Unclear	Unclear	Unclear
Jaspers Focks-2016	Low risk	Unclear	High risk	High risk	High risk
Steinberg-2016	Low risk	Unclear	High risk	Unclear	High risk
Poli-2017	Low risk	Unclear	Unclear	Unclear	Unclear
Caro Martínez-2017	Low risk	Unclear	Unclear	Unclear	Unclear

Esteve-Pastor-2017	Low risk	Unclear	Unclear	Unclear	Unclear
Rivera-Caravaca-2017	Low risk	Unclear	Unclear	Unclear	Unclear
Fox-2021	Low risk	Unclear	Unclear	Unclear	Unclear
Beshir-2018	Low risk	Unclear	High risk	High risk	High risk
Chao-2018	Low risk	Unclear	Unclear	Unclear	Unclear
Dalgaard-2019	Low risk	Unclear	High risk	Unclear	High risk
Lip-2018	Low risk	Unclear	Unclear	Unclear	Unclear
Mori-2019	Low risk	Unclear	High risk	Unclear	High risk
O'Brien-2015	Low risk	Unclear	Unclear	Unclear	Unclear
Quinn-2016	Low risk	Unclear	High risk	Unclear	High risk
Yao-2017	Low risk	Unclear	High risk	Unclear	High risk
Claxton-2018	Low risk	Unclear	High risk	Unclear	High risk
Elvira-Ruiz-2020	Low risk	Unclear	Unclear	Low risk	Unclear
Rutherford-2018	Low risk	Unclear	High risk	Unclear	High risk
Adam-2021	Low risk	Unclear	Unclear	Low risk	Unclear
Siu-2014	Low risk	Unclear	High risk	Unclear	High risk
Suzuki-2014	Low risk	Unclear	High risk	Unclear	High risk
Prochaska-2018	Low risk	Unclear	Unclear	Unclear	Unclear
Schwartz-2019	Low risk	Unclear	Unclear	Unclear	Unclear
Ravvaz-2021	Low risk	Unclear	High risk	Unclear	High risk

PROBAST=prediction model risk of bias assessment tool

Supplemental Table 5. Calibration analysis of the HAS-BLED score in the included studies

	**Calibration analysis**
Proietti-2018b	The ORBIT score had the best agreement over the range of bleeding risk when compared to the original derivation cohort. Conversely, the ATRIA showed the largest mismatch in calibration. The ATRIA and HAS-BLED tended to overestimate the risk of bleeding. The HEMORR2HAGES underestimated the risk of bleeding events, in particularly for those patients with a higher predicted risk.
Jaspers Focks-2016	HAS-BLED, HEMORR2HAGES, and ATRIA had nonsignificant P values indicating an adequate calibration
Beshir-2018	Adequate calibration
Lip-2018	Compared with the corresponding rates from the respective derivation cohorts, the rates were generally higher for ATRIA, lower for HAS-BLED whereas ORBIT appeared well-calibrated up to score 4.
Mori-2019	The ORBIT bleeding score showed a similar predictive performance compared with the HAS-BLED score (slope: 0.91 [95% CI 0.40, 1.43] vs 0.72 [95% CI 0.03, 1.40], intercept: 0.24 [95% CI 2.13, 2.61] vs 0.71 [95% CI 2.35, 3.76], respectively)
O'Brien-2015	The ORBIT score displayed superior calibration compared with the other 2 scores, followed by HAS-BLED (worst at low risk strata) and ATRIA (not good for most risk groups)
Yao-2017	ORBIT and HAS-BLED were reported to have better calibration than ATRIA

Supplemental Table 6. Decision curve analysis of the HAS-BLED score in the included studies

	Decision curve analysis
Proietti-2018a	**Any clinically relevant bleeding:**<br>No apparent net benefit of HAS-BLED versus GARFIELD-AF<br>**Major bleeding:**<br>A slight higher net benefit was observed for HAS-BLED than GARFIELD-AF<br>**Any bleeding:**<br>The HAS-BLED had a net benefit of ≈5% over the GARFIELD-AF bleeding score for any bleeding
Apostolakis-2012	**Any clinically relevant bleeding:**<br>The HAS-BLED was superior to the HEMORR2HAGES and ATRIA for any threshold probability
Apostolakis-2013	**Any clinically relevant bleeding:**<br>For threshold probabilities above 9%, HAS-BLED resulted in higher net benefit compare to CHADS2 and CHA2DS2-VASc
Hijazi-2016	**Major bleeding:**<br>ABC-bleeding risk score provided a larger net benefit across the range of major bleeding risk compared with both the HAS-BLED and ORBIT；<br>A higher net benefit was observed for ORBIT than HAS-BLED
Esteve-Pastor-2017	**Major bleeding:**<br>The use of HAS-BLED score implied an approximate net benefit of 4 % over the ABC-Bleeding score
Chao-2018	**Major bleeding:**<br>HAS-BLED had better net benefit of predicting major bleeding compared to the MBR factors score
Lip-2018	If the intervention threshold is low (<1.7%), the benefit is toward monitoring all patients. If preference is for a major bleeding risk threshold between 1.7% and 2.0%, most benefit was obtained by using HAS-BLED. The ORBIT and ATRIA scores provided better benefit for thresholds between 2.0% and 6.0%

Supplemental Table 7. Modified HAS-BLED in the published studies

Study (author-year)	Data source	Study design	Type of anticoagulants analyzed	Study endpoints	Modified HAS-BLED
Hijazi-2017	The RE-LY trial	Retrospective cohort	Dabigatran; warfarin	Major bleeding	HAS-BLED + GDF-15
Rivera-Caravaca-2019	Single anticoagulation centre in a tertiary hospital in Murcia, Spain; 2007	Prospective cohort	Acenocoumarol	Major bleeding	HAS-BLED + vWF + NT-proBNP + IL-6 + Troponin T + BTP + soluble fibrin monomer complex
Roldan-2018	Single anticoagulation centre in a tertiary hospital in Murcia, Spain; 2007	Prospective cohort	Acenocoumarol	Major bleeding	HAS-BLED + vWF + high sensitivity troponin T + NT-proBNP + high sensitivity IL-6 + TTR + modification of diet in renal disease
Serna-2018	Single anticoagulation centre in a tertiary hospital in Murcia, Spain; 2007	Prospective cohort	Acenocoumarol	Major bleeding	HAS-BLED + carrying VKORC1 allele and CYP2C9*3 polymorphisms
Prochaska-2018	The thrombEVAL cohort. Denmark	Prospective cohort	Phenprocoumon	Any clinically relevant bleeding	Simplified HAS-BLED (only the factors of age >65 years, history of major bleeding,and sustained AF in the presence of heart failure)
Elvira-Ruiz-2020	Two hospitals in Spain; 2013-2016	Retrospective cohort	DOACs; VKAs	Major bleeding	HAS-BLED + aortic stenosis
Ravvaz-2021	Longitudinal electronic health records in eastern Wisconsin and northern Illinois	Retrospective cohort	Warfarin	Any bleeding	HAS-BLED+area deprivation index
Chao-2018	National Health Insurance Research Database, Taiwan; 1998-2011	Retrospective cohort	Warfarin	Major bleeding	Baseline, Follow-up and Delta HAS-BLED

Suzuki-2014	Kameda Medical Center; Japan; 2005	Prospective cohort	Warfarin	Major bleeding	Modified HAS-BLED (renal dysfunction defined by eGFR <60, with exclusion of the 'elderly' factor because eGFR is calculated based on patient age)

Abbreviations: HAS-BLED=Hypertension, Abnormal liver/renal function, Stroke, Bleeding history or predisposition, Labile international normalized ratio, Elderly, Drugs/alcohol concomitantly.

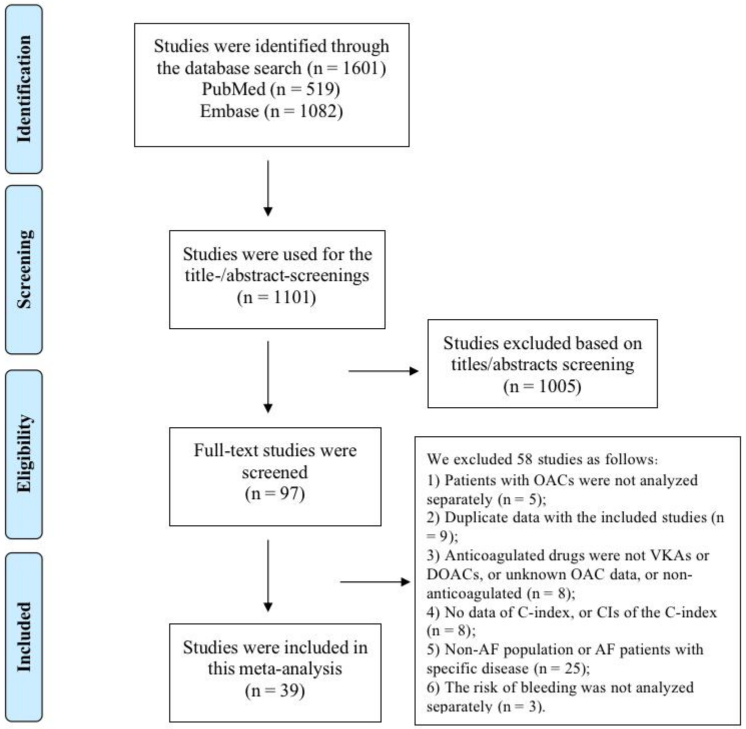

Supplementary Figure 1. Flow chart of document retrieval in this meta-analysis

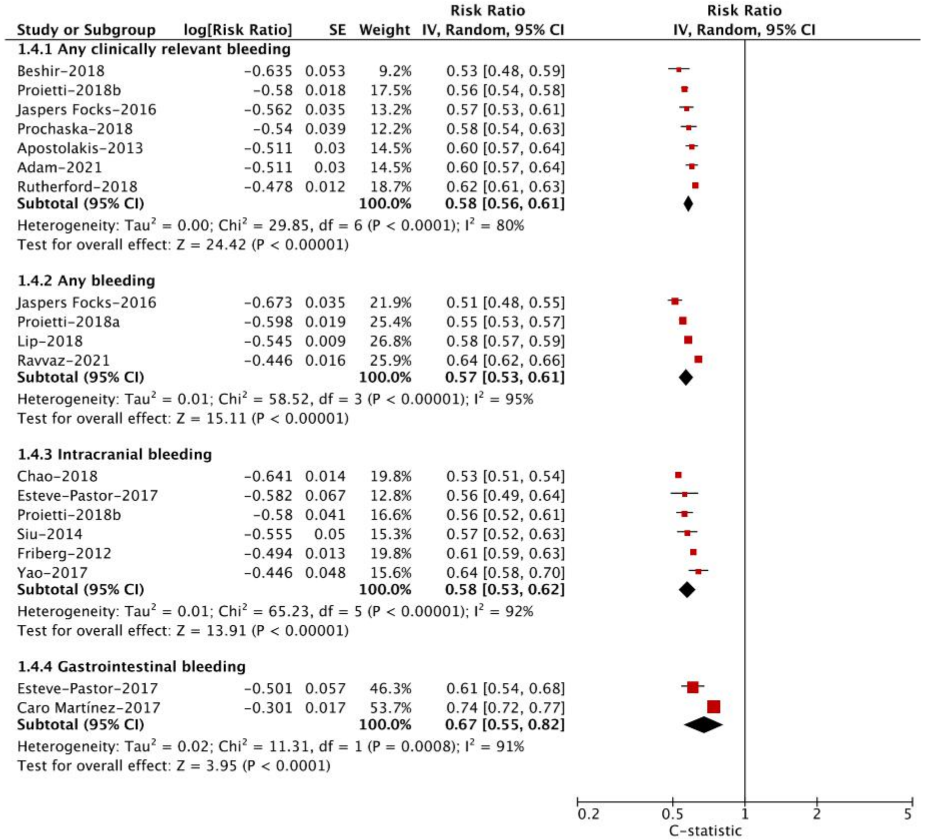

Supplementary Figure 2. The pooled analysis of the C-statistics for any clinically relevant bleeding, any bleeding, intracranial bleeding, and gastrointestinal bleeding in anticoagulated patients with atrial fibrillation

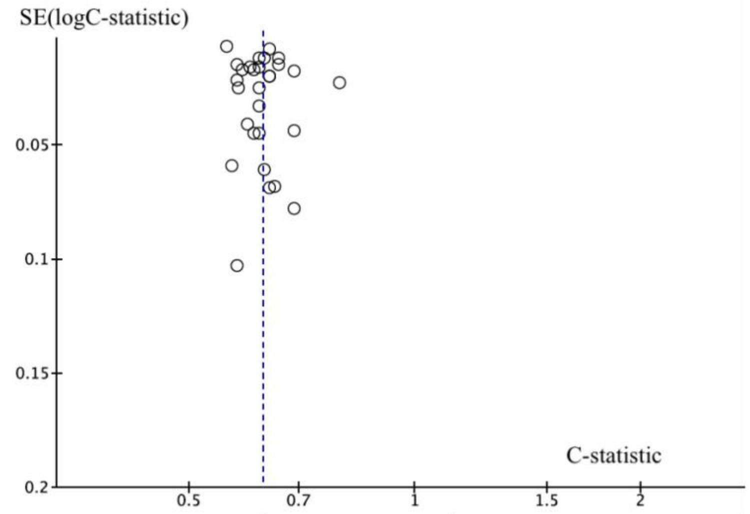

Major bleeding

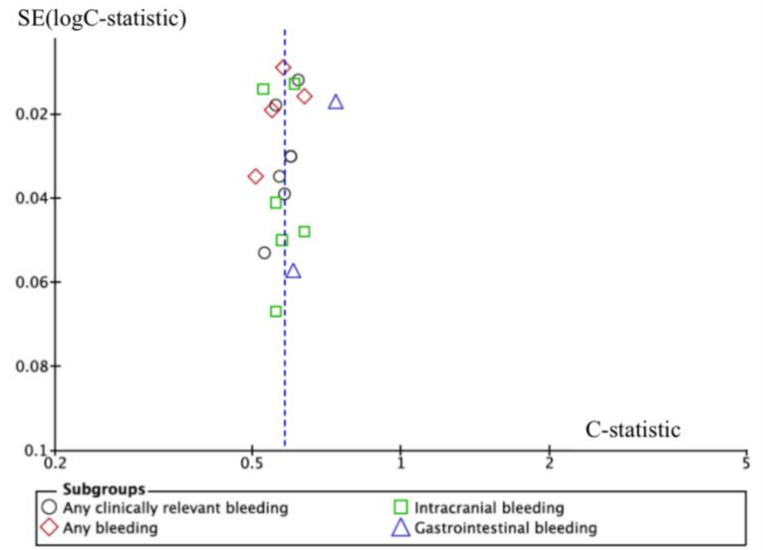

Supplementary Figure 3. The publication biases inspected by the funnel plots

Study or Subgroup	log[Risk Ratio]	SE	Weight	Risk Ratio IV, Random, 95% CI
**1.3.1 Post hoc analysis of RCT**				
Apostolakis-2012	-0.431	0.068	3.4%	0.65 [0.57, 0.74]
Berg-2019	-0.478	0.016	18.9%	0.62 [0.60, 0.64]
Hijazi-2016	-0.494	0.017	18.3%	0.61 [0.59, 0.63]
O'Brien-2015[ROCKET-AF]	-0.528	0.017	18.3%	0.59 [0.57, 0.61]
Proietti-2016	-0.543	0.015	19.6%	0.58 [0.56, 0.60]
Proietti-2018b	-0.478	0.012	21.5%	0.62 [0.61, 0.63]
**Subtotal (95% CI)**			**100.0%**	**0.61 [0.59, 0.62]**
Heterogeneity: Tau² = 0.00; Chi² = 17.33, df = 5 (P = 0.004); I² = 71%				
Test for overall effect: Z = 37.28 (P < 0.00001)				
**1.3.2 Prospective cohort**				
Barnes-2014	-0.371	0.044	7.8%	0.69 [0.63, 0.75]
Claxton-2018	-0.462	0.012	13.1%	0.63 [0.62, 0.65]
Esteve-Pastor-2016	-0.462	0.061	5.5%	0.63 [0.56, 0.71]
Esteve-Pastor-2017	-0.54	0.025	11.1%	0.58 [0.55, 0.61]
Jaspers Focks-2016	-0.562	0.059	5.8%	0.57 [0.51, 0.64]
Mori-2019	-0.478	0.045	7.6%	0.62 [0.57, 0.68]
O'Brien-2015[ORBIT-AF]	-0.446	0.02	11.9%	0.64 [0.62, 0.67]
Poli-2017	-0.494	0.045	7.6%	0.61 [0.56, 0.67]
Roldan-2013b	-0.371	0.018	12.3%	0.69 [0.67, 0.71]
Steinberg-2016	-0.503	0.016	12.6%	0.60 [0.59, 0.62]
Suzuki-2014	-0.446	0.069	4.7%	0.64 [0.56, 0.73]
**Subtotal (95% CI)**			**100.0%**	**0.63 [0.61, 0.65]**
Heterogeneity: Tau² = 0.00; Chi² = 50.28, df = 10 (P < 0.00001); I² = 80%				
Test for overall effect: Z = 25.04 (P < 0.00001)				
**1.3.3 Retrospective cohort**				
Beshir-2018	-0.545	0.103	4.8%	0.58 [0.47, 0.71]
Chao-2018	-0.576	0.007	10.4%	0.56 [0.55, 0.57]
Dalgaard-2019	-0.446	0.008	10.4%	0.64 [0.63, 0.65]
Elvira-Ruiz-2020	-0.416	0.015	10.2%	0.66 [0.64, 0.68]
Fox-2021	-0.478	0.033	9.3%	0.62 [0.58, 0.66]
Friberg-2012	-0.511	0.041	8.8%	0.60 [0.55, 0.65]
Olesen-2011	-0.229	0.023	9.9%	0.80 [0.76, 0.83]
Pisters-2010	-0.371	0.078	6.2%	0.69 [0.59, 0.80]
Quinn-2016	-0.446	0.02	10.0%	0.64 [0.62, 0.67]
Schwartz-2019	-0.545	0.022	9.9%	0.58 [0.56, 0.61]
Yao-2017	-0.416	0.012	10.3%	0.66 [0.64, 0.68]
**Subtotal (95% CI)**			**100.0%**	**0.64 [0.60, 0.68]**
Heterogeneity: Tau² = 0.01; Chi² = 385.04, df = 10 (P < 0.00001); I² = 97%				
Test for overall effect: Z = 14.80 (P < 0.00001)				

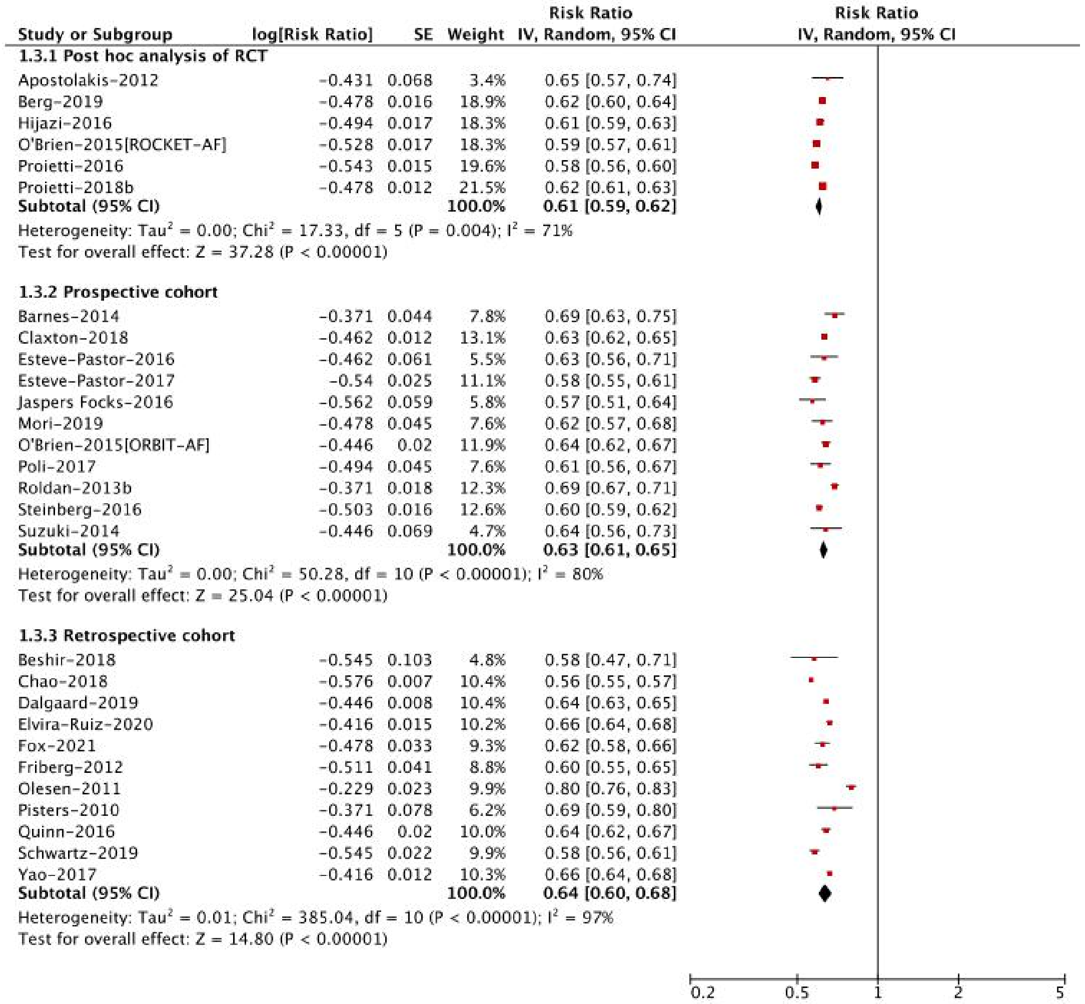

Test for subgroup differences: Chi² = 3.98, df = 2 (P = 0.14), I² = 49.7%

Supplementary Figure 4. Subgroup analysis for pooling the C-statistics of the HAS-BLED score based on the study design

Study or Subgroup	log[Risk Ratio]	SE	Weight	Risk Ratio IV, Random, 95% CI
**1.5.1 Labile INR:yes**				
Jaspers Focks-2016	−0.562	0.059	3.9%	0.57 [0.51, 0.64]
Proietti-2016	−0.543	0.015	15.1%	0.58 [0.56, 0.60]
Esteve-Pastor-2017	−0.54	0.025	11.2%	0.58 [0.55, 0.61]
Hijazi-2016	−0.494	0.017	14.3%	0.61 [0.59, 0.63]
Poli-2017	−0.494	0.045	5.8%	0.61 [0.56, 0.67]
Berg-2019	−0.478	0.016	14.7%	0.62 [0.60, 0.64]
Proietti-2018b	−0.478	0.012	16.3%	0.62 [0.61, 0.63]
Quinn-2016	−0.446	0.02	13.1%	0.64 [0.62, 0.67]
Suzuki-2014	−0.446	0.069	3.0%	0.64 [0.56, 0.73]
Pisters-2010	−0.371	0.078	2.5%	0.69 [0.59, 0.80]
**Subtotal (95% CI)**			**100.0%**	**0.61 [0.60, 0.63]**
Heterogeneity: $Tau^2 = 0.00$; $Chi^2 = 26.89$, df = 9 (P = 0.001); $I^2 = 67\%$				
Test for overall effect: Z = 37.67 (P < 0.00001)				
**1.5.2 Labile INRs:no**				
Chao-2018	−0.576	0.007	6.8%	0.56 [0.55, 0.57]
Poli-2017	−0.545	0.048	5.2%	0.58 [0.53, 0.64]
Schwartz-2019	−0.545	0.022	6.5%	0.58 [0.56, 0.61]
Beshir-2018	−0.545	0.103	2.8%	0.58 [0.47, 0.71]
O'Brien-2015[ROCKET-AF]	−0.528	0.017	6.6%	0.59 [0.57, 0.61]
Friberg-2012	−0.511	0.041	5.6%	0.60 [0.55, 0.65]
Steinberg-2016	−0.503	0.016	6.7%	0.60 [0.59, 0.62]
Fox-2021	−0.478	0.033	6.0%	0.62 [0.58, 0.66]
Claxton-2018	−0.462	0.012	6.8%	0.63 [0.62, 0.65]
Esteve-Pastor-2016	−0.462	0.061	4.5%	0.63 [0.56, 0.71]
Dalgaard-2019	−0.446	0.008	6.8%	0.64 [0.63, 0.65]
O'Brien-2015[ORBIT-AF]	−0.446	0.02	6.5%	0.64 [0.62, 0.67]
Apostolakis-2012	−0.431	0.068	4.2%	0.65 [0.57, 0.74]
Elvira-Ruiz-2020	−0.416	0.015	6.7%	0.66 [0.64, 0.68]
Roldan-2013b	−0.371	0.018	6.6%	0.69 [0.67, 0.71]
Barnes-2014	−0.371	0.044	5.4%	0.69 [0.63, 0.75]
Olesen-2011	−0.229	0.023	6.4%	0.80 [0.76, 0.83]
**Subtotal (95% CI)**			**100.0%**	**0.63 [0.60, 0.66]**
Heterogeneity: $Tau^2 = 0.01$; $Chi^2 = 409.98$, df = 16 (P < 0.00001); $I^2 = 96\%$				
Test for overall effect: Z = 20.85 (P < 0.00001)				

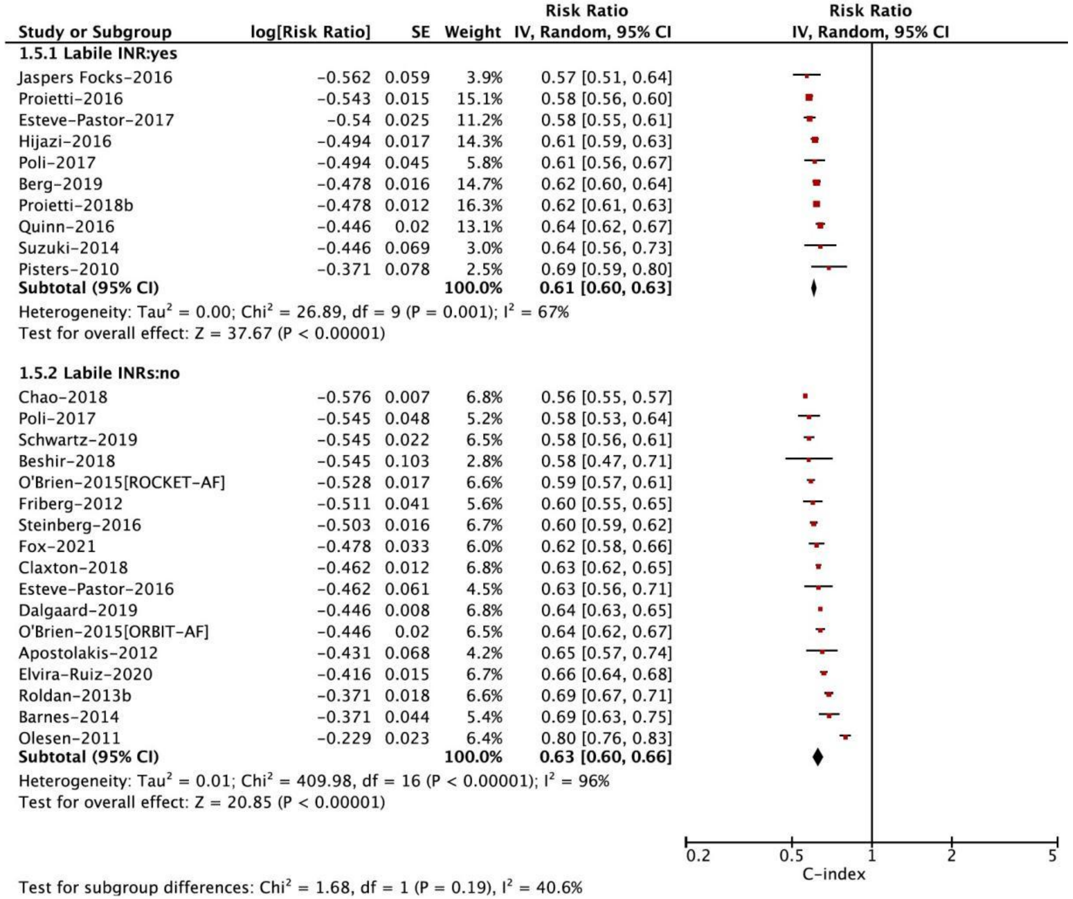

Test for subgroup differences: $Chi^2 = 1.68$, df = 1 (P = 0.19), $I^2 = 40.6\%$

Supplementary Figure 5. Subgroup analysis for pooling the C-statistics of the HAS-BLED score based on available vs. unavailable labile INRs

Study or Subgroup	log[Risk Ratio]	SE	Weight	Risk Ratio IV, Random, 95% CI
**1.4.1 ≤12 months**				
Barnes-2014	−0.371	0.044	8.0%	0.69 [0.63, 0.75]
Beshir-2018	−0.545	0.103	3.1%	0.58 [0.47, 0.71]
Claxton-2018	−0.462	0.012	12.1%	0.63 [0.62, 0.65]
Dalgaard-2019	−0.446	0.008	12.4%	0.64 [0.63, 0.65]
Esteve-Pastor-2016	−0.462	0.061	6.0%	0.63 [0.56, 0.71]
Mori-2019	−0.478	0.045	7.9%	0.62 [0.57, 0.68]
Olesen-2011	−0.229	0.023	10.9%	0.80 [0.76, 0.83]
Pisters-2010	−0.371	0.078	4.5%	0.69 [0.59, 0.80]
Quinn-2016	−0.446	0.02	11.3%	0.64 [0.62, 0.67]
Steinberg-2016	−0.503	0.016	11.7%	0.60 [0.59, 0.62]
Yao-2017	−0.416	0.012	12.1%	0.66 [0.64, 0.68]
**Subtotal (95% CI)**			**100.0%**	**0.65 [0.63, 0.68]**
Heterogeneity: $Tau^2 = 0.00$; $Chi^2 = 112.68$, df = 10 (P < 0.00001); $I^2 = 91\%$				
Test for overall effect: Z = 20.46 (P < 0.00001)				
**1.4.2 > 12 months**				
Apostolakis-2012	−0.431	0.068	3.4%	0.65 [0.57, 0.74]
Berg-2019	−0.478	0.016	6.8%	0.62 [0.60, 0.64]
Chao-2018	−0.576	0.007	7.1%	0.56 [0.55, 0.57]
Elvira-Ruiz-2020	−0.416	0.015	6.8%	0.66 [0.64, 0.68]
Esteve-Pastor-2017	−0.54	0.025	6.2%	0.58 [0.55, 0.61]
Fox-2021	−0.478	0.033	5.7%	0.62 [0.58, 0.66]
Friberg-2012	−0.511	0.041	5.1%	0.60 [0.55, 0.65]
Hijazi-2016	−0.494	0.017	6.7%	0.61 [0.59, 0.63]
Jaspers Focks-2016	−0.562	0.059	3.9%	0.57 [0.51, 0.64]
O'Brien-2015[ORBIT-AF]	−0.446	0.02	6.5%	0.64 [0.62, 0.67]
O'Brien-2015[ROCKET-AF]	−0.528	0.017	6.7%	0.59 [0.57, 0.61]
Poli-2017	−0.494	0.045	4.8%	0.61 [0.56, 0.67]
Proietti-2016	−0.543	0.015	6.8%	0.58 [0.56, 0.60]
Proietti-2018b	−0.478	0.012	6.9%	0.62 [0.61, 0.63]
Roldan-2013b	−0.371	0.018	6.7%	0.69 [0.67, 0.71]
Schwartz-2019	−0.545	0.022	6.4%	0.58 [0.56, 0.61]
Suzuki-2014	−0.446	0.069	3.4%	0.64 [0.56, 0.73]
**Subtotal (95% CI)**			**100.0%**	**0.61 [0.59, 0.63]**
Heterogeneity: $Tau^2 = 0.00$; $Chi^2 = 223.25$, df = 16 (P < 0.00001); $I^2 = 93\%$				
Test for overall effect: Z = 28.35 (P < 0.00001)				

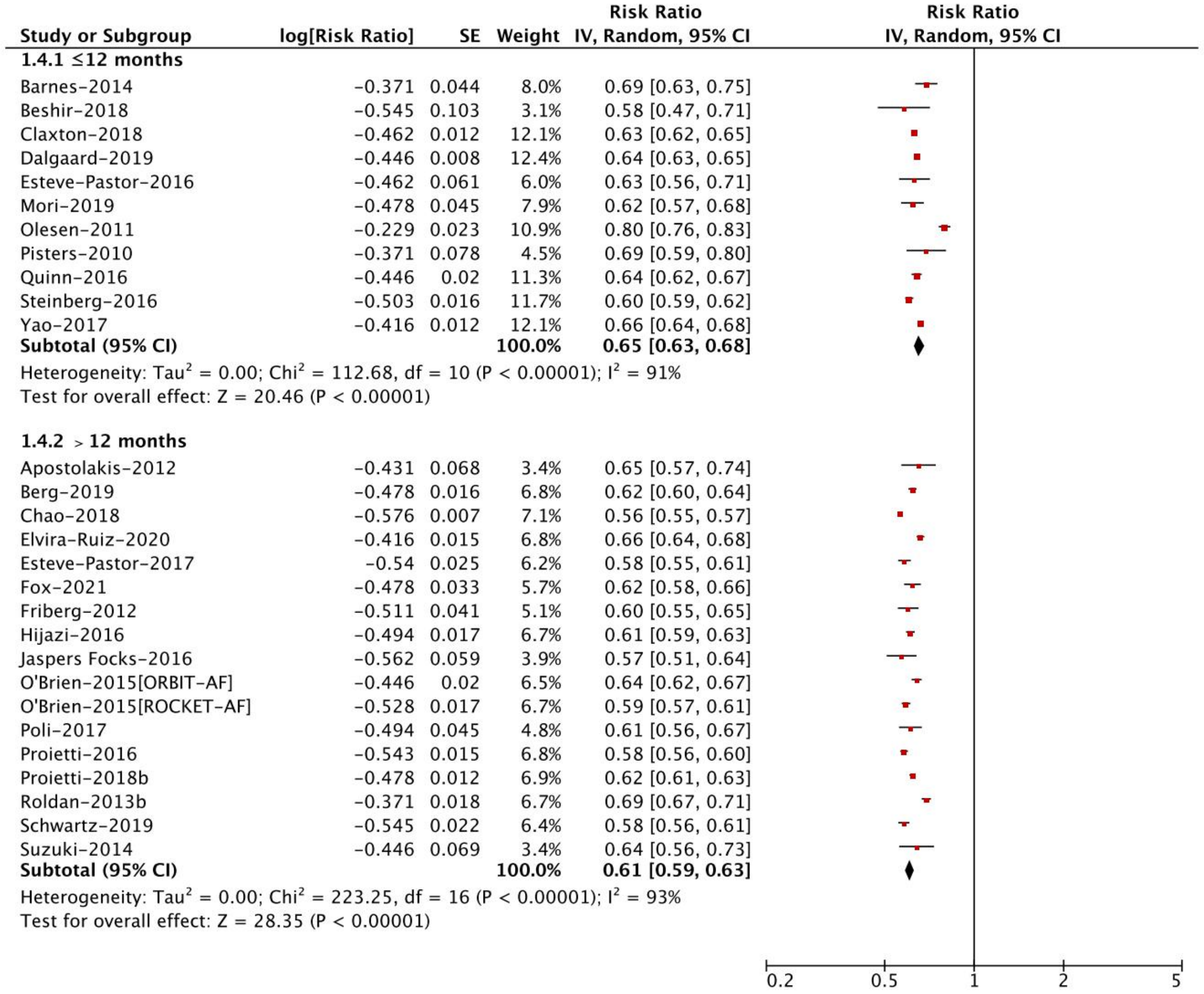

Test for subgroup differences: $Chi^2 = 6.17$, df = 1 (P = 0.01), $I^2 = 83.8\%$

Supplementary Figure 6. Subgroup analysis for pooling the C-statistics of the HAS-BLED score based on the follow-up time
